# Volatile 2‐Phenylethanol and β‐Cyclocitral Trigger Defense‐Related Transcriptional and Metabolic Changes in Grapevine Leaves Against Downy Mildew

**DOI:** 10.1111/ppl.70412

**Published:** 2025-07-22

**Authors:** Sara Avesani, Valentina Lazazzara, Matteo Buti, Michael Oberhuber, Peter Robatscher, Michele Perazzolli

**Affiliations:** ^1^ Center for Agriculture Food Environment (C3A) University of Trento San Michele all’Adige Italy; ^2^ Laboratory for Flavours and Metabolites Laimburg Research Centre Auer (Ora) Italy; ^3^ Research and Innovation Centre Fondazione Edmund Mach San Michele all’Adige Italy; ^4^ Department of Agriculture, Food, Environmental and Forestry Sciences (DAGRI) University of Florence Florence Italy

**Keywords:** induced resistance, transcriptomic analysis, untargeted metabolomics analysis, *Vitis vinifera*, volatile organic compounds

## Abstract

Volatile organic compounds (VOCs) are produced by grapevine leaves in response to phytopathogen infection. In particular, 2‐phenylethanol and β‐cyclocitral were triggered by *Plasmopara viticola* inoculation in downy mildew‐resistant genotypes, but no information is available on their involvement in plant resistance induction. This study aimed to clarify transcriptional and metabolic changes associated with VOC‐induced resistance activated by 2‐phenylethanol and β‐cyclocitral treatments against *P. viticola* . Both VOCs decreased downy mildew severity on grapevine leaf disks by complex reprogramming of the grapevine transcriptome at 1 and 6 days post inoculation. RNA‐Seq analysis showed the modulation of transcripts related to defense (chitinases, defense‐related proteins, and pathogenesis‐related proteins), oxidative stress (glutathione S‐transferases and peroxidases), secondary metabolism (nitrilases, stilbene synthases, and terpene synthases), signal transduction (e.g., kinases, receptor kinases, and calmodulins), and transcription (bHLH, ERF, MYB, NAC, and WRKY transcription factors) in leaf disks treated with 2‐phenylethanol and β‐cyclocitral. VOC treatments also caused changes in the leaf metabolome, and pathway analysis of metabolic features with significant changes in abundance in 2‐phenylethanol‐ and β‐cyclocitral‐treated leaf disks revealed the reprogramming of amino acid, carbohydrate, flavonoid, phenylpropanoid, and terpenoid metabolism. In particular, compounds with increases in abundance belonged to putative carbohydrates, carboxylic acids, and phenylpropanoids in 2‐phenylethanol‐treated samples, or carboxylic acids and terpenoids in β‐cyclocitral‐treated samples, including molecules possibly associated with plant defense against pathogens, such as 3‐dehydroquinic acid, 4‐thujanol, aromadendrin, camphor, *p*‐coumaryl alcohol, and perillaldehyde. Correlation analysis between transcriptomic and metabolomic data indicated that 2‐phenylethanol and β‐cyclocitral act as resistance inducers against downy mildew in grapevine.

## 1 Introduction

Plant volatile organic compounds (VOCs) act as bioactive molecules that rapidly diffuse to their biological targets (Adebesin et al. 2017; Widhalm et al. 2015). VOCs are synthesized in response to abiotic and biotic stimuli (Midzi et al. 2022) through different metabolic pathways (e.g., methylerythritol phosphate pathway, mevalonic acid pathway, shikimate pathway, phenylalanine pathway, and lipoxygenase pathway), and they can be classified as amino acid derivatives, benzenoids, fatty acid derivatives, phenylpropanoids, and terpenoids (Dudareva et al. 2013). Plant VOCs play crucial roles in plant communication with other organisms and plant defense against pathogens by directly inhibiting phytopathogen growth or inducing resistance (Quintana‐Rodriguez et al. 2015). For example, some VOCs (e.g., 2‐phenylethanol, carvacrol, caryophyllene, farnesene, limonene, linalool, and nonanal) can directly inhibit the growth of plant pathogens (Camacho‐Coronel et al. 2020; Huang et al. 2012; Lee et al. 2016; Neri et al. 2007; Quintana‐Rodriguez et al. 2015; Zou et al. 2023). Moreover, VOCs (e.g., β‐cyclocitral, caryophyllene, ionone, camphene, hexenal, isoprene, limonene, linalool, and pinene) can induce defense‐related processes against pathogens in different plant species (Avesani et al. 2023; Brambilla et al. 2022; Deshpande et al. 2021; Frank et al. 2021; Quintana‐Rodriguez et al. 2015; Riedlmeier et al. 2017; Rosenkranz et al. 2021; Taniguchi et al. 2023; Vlot et al. 2021). However, transcriptional and metabolic responses activated by VOC‐induced resistance against pathogens have not been investigated in perennial crops.

Grapevine ( *Vitis vinifera* ) is one of the most widespread perennial crops, and VOCs are known to be responsible for berry aroma and quality (Rienth et al. 2021). Grapevine VOCs are also synthesized in response to abiotic and biotic stresses, and VOCs belonging to the classes of alcohols, aldehydes, benzenoids, and terpenoids were found in response to downy mildew caused by *Plasmopara viticola* infection (Lazazzara et al. 2022). VOCs have been associated with downy mildew resistance in several grapevine genotypes, such as bc4, Kober 5BB, SO4, Solaris (Lazazzara et al. 2018), Bianca (Chitarrini et al. 2017; Ricciardi et al. 2021), a genotype resulting from the Bianca × SK77‐4/5 cross (Chitarrini et al. 2020), and Croatian cultivars (e.g., Malvazija istarska, Ranfol, and Teran) (Štambuk et al. 2023). In particular, 2‐phenylethanol (a volatile benzenoid also known as phenylethyl alcohol or phenethyl alcohol) and β‐cyclocitral (a volatile terpenoid) were found in Bianca (Chitarrini et al. 2017; Ricciardi et al. 2021), Croatian cultivars (Štambuk et al. 2023), Kober 5BB, SO4, Solaris (Lazazzara et al. 2018), and a pyramided genotype (Chitarrini et al. 2020), suggesting a role in grapevine defense mechanisms against downy mildew. 2‐phenylethanol and β‐cyclocitral decreased downy mildew symptoms on susceptible grapevine leaves (Lazazzara et al. 2018). Moreover, 2‐phenylethanol showed inhibitory activities against several phytopathogens, such as *Aspergillus flavus* (Hua et al. 2014), *Botrytis cinerea* (Huang et al. 2011; Zou et al. 2023), *Colletotrichum camelliae Massea* (Zhang et al. 2006), *Fusarium graminearum* (Sun et al. 2023), *Penicillium* sp. (Liu et al. 2014), *Rhizoctonia solani* (Kai et al. 2007), and *Rhizoctonia solanacearum* (Zhu et al. 2011). Likewise, β‐cyclocitral showed inhibitory effects against *Chlorella pyrenoidosa* (Ikawa et al. 2001), *Cyanobacterium microcystis* (Ozaki et al. 2009), and *Chlamydomonas reinhardtii* (Sun et al. 2020) cells. In addition to direct inhibitory effects, 2‐phenylethanol can modulate the expression of genes related to defense processes, phenylpropanoid biosynthesis, mitochondrial activity, cellular signaling, and hormone communication in orange fruits (Liu et al. 2016), as well as defense‐related genes in tomato plants (Kumar et al. 2024). Likewise, β‐cyclocitral can induce resistance mechanisms in rice against *Xanthomonas oryzae* pv. *oryzae*, upregulating the expression of defense‐related genes (Taniguchi et al. 2023). β‐cyclocitral is also known to upregulate the expression of genes related to abiotic and biotic stress responses (Deshpande et al. 2021) and to increase the abundance of compounds associated with plant growth and defense (e.g., amino acid and phenylpropanoid metabolism) in tomato leaves (Deshpande and Mitra 2023). β‐cyclocitral can affect abiotic stress tolerance (salt and water stress), (photo)oxidative stress tolerance, and root development in plants (Faizan et al. 2022; Havaux 2020) by the accumulation of salicylic acid‐ and *NPR1*‐dependent transcriptional reprogramming of glutathione‐S‐transferase genes (Lv et al. 2015). Moreover, β‐cyclocitral can alleviate cadmium toxicity by upregulating transport‐related genes in quinoa seedlings (Sun et al. 2021), suggesting transcriptional and metabolic responses to VOC treatments. However, no information is available on cellular responses activated by 2‐phenylethanol and β‐cyclocitral in grapevine tissues. This study aimed to clarify transcriptional and metabolic changes associated with VOC‐induced resistance activated by 2‐phenylethanol and β‐cyclocitral treatments against *P. viticola* .

## 2 Materials and Methods

### 2.1 VOC Treatments, *Plasmopara viticola* Inoculation, and Sample Collection

Downy mildew‐susceptible grapevine plants ( *V. vinifera* cultivar Pinot Noir) were grown as previously described (Lazazzara et al. 2021), and the *P. viticola* population was maintained on grapevine plants by subsequent inoculations to obtain the *P. viticola* inoculum (2.5 × 10^5^ sporangia mL^−1^) (Lazazzara et al. 2021).

For VOC treatments, leaf disks (25 mm diameter) were obtained from the greenhouse‐grown grapevine plants (from the fourth to sixth node of each shoot). Leaf disks were placed on wet filter paper in a dish (dish chamber) with the abaxial surface uppermost (six leaf disks for each dish), as previously described (Avesani et al. 2023). Pure 2‐phenylethanol (CAS No. 60‐12‐8, Sigma‐Aldrich, Merck), β‐cyclocitral (CAS No. 432‐25‐7, Sigma‐Aldrich, Merck), and water (control) were 10‐fold diluted in dimethyl sulfoxide (DMSO; Sigma‐Aldrich, Merck), and each stock solution (50 μL VOC, or water, and 450 μL DMSO) was serially diluted in distilled water. Treatments were applied to a filter paper disk (Whatman, Merck) fixed on the lid (without physical contact with the leaf tissue) of a dish containing grapevine leaf disks, as previously described (Avesani et al. 2023), to obtain the concentration of 20 mg L^−1^ of air volume in the dish chamber (assuming the complete VOC evaporation from the filter paper). This concentration was previously optimized for 2‐phenylethanol and β‐cyclocitral treatment as the optimal dosage that allowed efficient downy mildew control with no phytotoxic effects on leaf disks (Lazazzara et al. 2018). Dishes were sealed with Parafilm (Beims) and incubated in the dark at 25°C ± 1°C for 24 h (Lazazzara et al. 2018). Leaf disks were inoculated with five drops (5 μL each) of a *P. viticola* suspension ( *P. viticola* ‐inoculated) or with five drops (5 μL each) of distilled water (mock‐inoculated). The respective treatment (2‐phenylethanol, β‐cyclocitral, or water diluted in DMSO and water) was applied again to the filter paper disk immediately after inoculation; dishes were sealed with Parafilm and incubated overnight (16 h) in the dark at 25°C ± 1°C. Dishes were dried under a laminar hood and incubated for 6 days under greenhouse conditions without Parafilm. This application procedure was chosen to maximize the VOC efficacy against downy mildew since 2‐phenylethanol and β‐cyclocitral were partially less active when applied only before inoculation or only immediately after inoculation (data not shown).

Downy mildew severity, disease reduction (efficacy), and phytotoxic effects were assessed at 6 days post inoculation (dpi), as previously described (Avesani et al. 2023). Briefly, downy mildew severity was assessed as a percentage of the leaf disk surface covered by sporulation (EPPO 2001), calculated as the sum of the five inoculum drops scored as follows: 0%, no sporulation; 10%, scarce sporulation; 20%, dense sporulation (Lazazzara et al. 2021). The disease severity of each replicate (dish) was then calculated as the average of the disease severity of leaf disks contained in the dish. The efficacy was calculated according to the following formula: (disease severity of control leaf disks—disease severity of VOC‐treated leaf disks)/disease severity of control leaf disks × 100. The presence of phytotoxic effects was assessed visually by checking for discoloration, chlorosis, and whitening of leaf disks (EPPO 2014). For the disease assessment, 20 replicates (dishes with six leaf disks each) were assessed for each treatment, and data were analyzed using Past 4.03 software (https://www.nhm.uio.no/english/research/resources/past/) to detect significant differences among treatments according to the Kruskal‐Wallis test with Dunn's post hoc test (*p* ≤ 0.05).

Leaf disks treated with 2‐phenylethanol or β‐cyclocitral (diluted in DMSO and water) and control leaf disks (treated with DMSO in water) were collected at 1 dpi and 6 dpi from *P. viticola* ‐inoculated and mock‐inoculated samples, and leaf disks were cut to 18 mm in diameter to eliminate areas where defense responses related to wounding are possible (Adrian et al. 2017). These time points were chosen to analyze the grapevine defense reactions (Avesani et al. 2023; Malacarne et al. 2011; Perazzolli et al. 2012; Vrhovsek et al. 2012). Three and six replicates were obtained for each treatment and time point for transcriptomic and metabolomic analysis, respectively, and each replicate comprised 10 leaf disks. Samples were immediately frozen in liquid nitrogen, crushed using a mixer mill disruptor (MM200, Retsch) at 25 Hz for 60 s with refrigerated 2 mL‐tubes and 6 mm‐beads, and stored at −80°C until further use.

### 2.2 RNA Extraction and Sequencing

Total RNA was extracted from an aliquot of each crushed sample (80 mg of leaf disk powder) using the Spectrum Plant Total RNA kit (Sigma‐Aldrich, Merck) with an on‐column DNase treatment with the RNase‐Free DNase Set (Qiagen). Total RNA was quantified using a Qubit RNA Broad range Assay Kit (Thermo Fisher Scientific), and RNA quality was checked using a High Sensitivity RNA ScreenTape kit and TapeStation 4150 instrument (Agilent Technologies). The effectiveness of the DNase treatment was confirmed by running a PCR with primers of the grapevine *actin* gene (Table S1), and no amplification signals were detected in the absence of reverse transcription.

Thirty‐six RNA samples [three treatments (control, 2‐phenylethanol‐treated, β‐cyclocitral‐treated), two inoculation conditions ( *P. viticola* ‐inoculated and mock‐inoculated), and two time points (1 and 6 dpi) with tree replicates (pool of 10 leaf disks each)] were subjected to RNA‐Seq library construction, using the TruSeq Stranded Total RNA Library Preparation kit (Illumina) and rRNA depletion with the RiboZero rRNA Removal Kit for plant (Illumina) according to the manufacturer's instructions. Paired‐end reads of 150 nucleotides were obtained using a NovaSeq instrument (Illumina) at Fasteris (Plan‐les‐Ouates).

### 2.3 RNA‐Seq Data Elaboration and Identification of Differentially Expressed Transcripts

Quality of RNA‐Seq reads was assessed with FastQC v0.11.5 (http://www.bioinformatics.babraham.ac.uk/projects/fastqc), adapter sequences and low‐quality reads were removed using Trimmomatic v0.39 (ILLUMINACLIP:TruSeq_PE.txt:2:30:10 LEADING:3 TRAILING:3 SLIDINGWINDOW:4:15 MINLEN:50) (Bolger et al. 2014). Filtered read pairs were mapped to the *V. vinifera* PN40024.v4 genome (https://integrape.eu/resources/genes‐genomes/genome‐accessions) (Velt et al. 2023) using HiSat2 v2.2.1 (Kim et al. 2019), and read counts were assessed using featureCounts v1.6.0 software (Liao et al. 2014) with default parameters, based on “exon” feature and “transcript_id” meta‐feature of PN40024.v4.1 grapevine gene predictions (Velt et al. 2023). Raw count data elaboration, multi‐dimensional scaling (MDS) plot, and differential expression analyses were carried out using the Bioconductor EdgeR v3.38.4 package (Robinson et al. 2009). Non‐active transcripts were filtered out [a transcript was considered active if reads per million mapping to that transcript (RPM) were > 1 in at least two libraries], and read counts were normalized according to library dimension. Differential expression analysis was carried out with the likelihood ratio test for seven pairwise comparisons for each timepoint (1) control *P. viticola* ‐inoculated and control mock‐inoculated samples; (2) 2‐phenylethanol‐treated *P. viticola* ‐inoculated and 2‐phenylethanol‐treated mock‐inoculated samples; (3) 2‐phenylethanol‐treated mock‐inoculated and control mock‐inoculated samples; (4) 2‐phenylethanol‐treated *P. viticola* ‐inoculated and control *P. viticola* ‐inoculated samples; (5) β‐cyclocitral‐treated *P. viticola* ‐inoculated and β‐cyclocitral‐treated mock‐inoculated samples; (6) β‐cyclocitral‐treated mock‐inoculated and control mock‐inoculated samples; (7) β‐cyclocitral‐treated *P. viticola* ‐inoculated and control *P. viticola* ‐inoculated samples. Differentially expressed transcripts (DETs) were selected, imposing a Log_2_‐transformed fold change (LFC) lower than −2 or higher than 2 and a false discovery rate (FDR) lower than 0.05. Venn diagram representations of DETs were visualized using a web tool (http://bioinformatics.psb.ugent.be/webtools/Venn/).

### 2.4 Functional Annotation of Differentially Expressed Transcripts

Protein descriptions and gene ontology (GO) annotations of each DET were obtained from the grapevine annotation file (PN40024.v4.1.REF.b2g.annot; https://integrape.eu/resources/genes‐genomes/genome‐accessions) (Velt et al. 2023). For each time point, DETs were grouped in transcripts with a direct defense profile (upregulation or downregulation in the pairwise comparisons between 2‐phenylethanol‐ or β‐cyclocitral‐treated compared to control leaf disks in both mock‐inoculated and *P. viticola* ‐inoculated samples) or an ISR‐specific profile (upregulation or downregulation in the pairwise comparisons between 2‐phenylethanol‐ or β‐cyclocitral‐treated compared to control leaf disks in *P. viticola* ‐inoculated samples and not modulated in mock‐inoculated samples). Moreover, no DETs with priming profiles were found, such as transcripts with a reinforced modulation in VOC‐treated *P. viticola* ‐inoculated samples compared to control *P. viticola* ‐inoculated samples. Direct defense and ISR‐specific transcripts were classified into 14 functional categories (carbohydrate metabolism, defense, energy metabolism, growth and development, hormone metabolism, lipid metabolism, metabolism, oxidative stress, protein and amino acid metabolism, secondary metabolism, signal transduction, transcription, transport, and uncharacterized) according to the manually curated annotation based on the protein description search on the UniProtKB database (https://www.uniprot.org/) and GO biological process annotations of QuickGO (https://www.ebi.ac.uk/QuickGO/). Heatmaps summarizing expression profiles and putative functions of DETs belonging to functional categories possibly involved in grapevine resistance against downy mildew (defense, oxidative stress, secondary metabolism, signal transduction, and transcription) (Azevedo et al. 2022; Gong et al. 2022; Vigneron et al. 2023) were obtained with the R package *pheatmap* (version 1.0.12) (https://CRAN.R‐project.org/package=pheatmap.2019).

### 2.5 Gene Expression Analysis by Quantitative Real‐Time RT‐PCR

Synthesis of cDNA and quantitative real‐time PCR (qPCR) reactions were carried out using specific primers (Table S1) with the Light Cycler 480 (Roche Diagnostics), as previously described (Avesani et al. 2023). Genes encoding chitinase 3 (*CHIT‐3*), lipoxygenase 9 (*LOX‐9*), osmotin 2 (*OSM‐2*), pathogenesis‐related (PR) protein 2 (*PR‐2*) and protein 4 (*PR‐4*) were used as markers of grapevine‐induced resistance against downy mildew (Banani et al. 2014; Perazzolli et al. 2012), markers of salicylic acid (SA) defense pathways (*PR‐2*) (Gauthier et al. 2014), and jasmonic acid (JA) defense pathways (*PR‐4* and *LOX‐9*) (Hamiduzzaman et al. 2005). The hypersensitive response‐related gene (*HSR*) was used as a marker of cell death (Lakkis et al. 2019) and VOC‐induced resistance (Lazazzara et al. 2021), while the stilbene synthase gene (*STS*) was used as a marker of the phenylpropanoid pathway (Lakkis et al. 2019). Cycle threshold values were calculated with the Light Cycler 480 SV 1.5.0 software according to the second derivative calculation, and reaction efficiencies were calculated with the LinRegPCR 11.1 software (Ruijter et al. 2009). The expression level of each gene was calculated according to the Hellemans equation (Hellemans et al. 2007), and normalized relative quantities were calculated with respect to control mock‐inoculated samples at each time point, using *actin* and *VATP16* as housekeeping genes (Avesani et al. 2023). The determination coefficient R^2^ between gene expression levels assessed by RNA‐Seq and qPCR analysis was calculated using Microsoft Excel (Microsoft Corporation).

### 2.6 Metabolomic Analysis by Ultra High Pressure Liquid Chromatography—Heated Electrospray Ionization—Orbitrap Mass Spectrometry (UHPLC‐HESI‐Orbitrap‐MS)

An aliquot of each crushed sample (30 mg of leaf disk powder) was supplemented with an internal standard mixture (20 μL), consisting of 1 g L^−1^ caffeine‐(trimethyl‐d9) (Sigma‐Aldrich, Merck) and 1 g L^−1^ choline chloride‐(trimethyl‐d9) (Sigma‐Aldrich, Merck) to validate the extraction efficiency, and metabolite extraction was carried out as reported by Avesani et al. (2023). Briefly, samples were extracted in 1 mL of methanol:water (80:20; v v^−1^) with sonication for 15 min (Ultrasonic Cleaners, VWR) and shaking for 15 min (Thermomixer, Eppendorf) at 4°C. Samples were centrifuged at 20,000 *g* for 15 min at 4°C, the supernatant was collected and stored at 4°C overnight. Before chromatographic analyses, sample extracts were centrifuged again at 20,000 *g* for 15 min at 4°C, and each vial was prepared by mixing an aliquot of the sample extract (240 μL) with an additional internal standard mixture (10 μL) consisting of (+)‐catechin‐2,3,4‐^13^C_3_ (Sigma‐Aldrich, Merck; 500 mg L^−1^), and (±)‐catechin‐2,3,4‐^13^C_3_ gallate (Sigma‐Aldrich, Merck; 500 mg L^−1^), in order to monitor instrument performance and signal stability. As quality control (QC) samples, equal aliquots of each sample extract were mixed and used to assess technical variability. Samples were analyzed in a randomized complete block design, and a QC sample was assessed every five samples.

Ultra high pressure liquid chromatography—heated electrospray ionization—Orbitrap mass spectrometry (UHPLC‐HESI‐Orbitrap‐MS) analysis was carried out using a Vanquish Flex UHPLC System (Thermo Scientific) coupled with an Orbitrap Exploris 240 mass spectrometer (Thermo Scientific). A Waters Acquity HSS T3 C18 column (150 × 2.1 mm, 1.8 μm; Waters Corporation) was used to separate metabolic analytes (injection volume of 5 μL) with a flow rate of 0.4 mL min^−1^ at 35°C. The mobile phase consisted of 0.1% formic acid (Sigma‐Aldrich, Merck) in water (solvent A) and 0.1% formic acid in acetonitrile (solvent B, Sigma‐Aldrich, Merck). The chromatographic separation was performed as reported by Šuković et al. (2020), with slight modifications. Briefly, a linear gradient program was set as follows: 5% solvent B in the first 1.0 min, increment from 5% to 99% solvent B from 1.0 min to 14.0 min, 99% solvent B until 19.0 min, decrement from 99% to 5% solvent B until 19.5 min, and 5% solvent B until 26 min. The ion source parameters were set according to Zhou et al. (2022) as follows: spray voltage was 3500 V in positive HESI mode and 2500 V in negative HESI mode; the temperature of the ion source, capillary, and the auxiliary gas was 350°C, 300°C, and 300°C, respectively; the setting of aux gas, sweep gas, and sheath gas was 10 arb, 0 arb, and 50 arb, respectively; the S‐lens RF value was 50 V; isolation window was 2 *m/z*; automatic gain control target was 2.0 × 10^5^; the maximum injection time was 100 ms. Mass spectrometric conditions were set according to Adobor et al. (2023), with slight modifications. In particular, the mass‐to‐charge ratio (*m/z*) range for the full scan mass spectrometry (MS) analysis was 90–1350 with a resolution of 180,000, and data‐dependent tandem mass spectrometry (ddMS2) fragmentation was acquired separately in each ionization mode at 20, 40, and 60 normalized collision energies with a resolution of 45,000, using the AcquireX data acquisition workflow (Thermo Scientific). The extraction efficiency was confirmed by calculating the extraction yield of internal standards added to each sample powder (greater than 90%), and the signal stability was confirmed by the analysis of relative standard deviations of internal standards added in each sample extract (lower than 5%), as previously reported (Avesani et al. 2023).

### 2.7 Metabolomic Data Processing and Selection of Metabolic Features With Significant Changes in Abundance

Full scan MS data and ddMS2 fragmentation data were processed from 72 samples [three treatments (control, 2‐phenylethanol‐treated, β‐cyclocitral‐treated), two inoculation conditions ( *P. viticola* ‐inoculated and mock‐inoculated), and two time points (1 and 6 dpi) with six replicates (pool of 10 leaf disks each)] using an untargeted metabolomics workflow (Untargeted Metabolomics with Statistics Detect Unknown with ID using Online Database and mzLogic) on the Compound Discoverer software (Version 3.3 SP2; Thermo Scientific) described by Lang et al. (2019), with slight modifications. The workflow involved spectrum selection, chromatographic alignment, metabolic features detection, and peak area normalization according to QC samples (QC correction) to detect metabolic features (detected metabolic features; Figure S1). A principal component analysis (PCA) was carried out on detected metabolic features using the MetaboAnalyst online platform (version 6.0; http://www.metaboanalyst.ca/) (Pang et al. 2022) with interquartile range (IQR) data filtering on peak areas, data normalization according to QC samples, Log_10_ transformation, and Pareto scaling as previously described (Avesani et al. 2023).

Metabolic features with significant changes in abundances were selected with the Compound Discoverer software (Version 3.3 SP2), imposing an LFC lower than −1 or higher than 1 and a *p* value of *t*‐test lower than 0.05 in seven pairwise comparisons for each time point, as described above for differential expression analysis.

### 2.8 Chemical Annotation of Metabolic Features

Metabolic features were annotated with the Compound Discoverer software (Version 3.3 SP2; Thermo Scientific) workflow (Figure S1). Briefly, putative chemical names and elemental formulas were obtained (annotated metabolic features) by searching in the Human Metabolome Database (HMDB; https://hmdb.ca/), Lipid Maps (https://www.lipidmaps.org/), MassBank (https://massbank.eu/MassBank/), Kyoto Encyclopedia of Genes and Genomes (KEGG; https://www.kegg.jp/), FooDB (https://foodb.ca/), PlantCyc (https://www.plantcyc.org/), ChEBI (https://www.ebi.ac.uk/chebi/), Phenol‐Explorer (http://phenol‐explorer.eu/), and Arita lab (6549 flavonoid structures; http://metabolomics.jp/wiki/) databases. The most probable chemical name, delta mass error (with a maximum mass error acceptance of 3 ppm), molecular weight, reference ion, fragmentation information, and neutral losses were obtained with the Compound Discoverer software (Version 3.3 SP2) workflow (Figure S1). All putative chemical names (Number of ChemSpider and mzCloud results) found by the Compound Discoverer software (Version 3.3 SP2) were reported for each annotated metabolic feature. Annotated metabolic features with significant increases and decreases in abundance were grouped for each time point according to Venn diagrams obtained with a web tool (http://bioinformatics.psb.ugent.be/webtools/Venn/).

To complete the chemical annotation, entry codes of the KEGG database were obtained with the peak annotation tool of MetaboAnalyst 6.0 online platform (version 6.0; http://www.metaboanalyst.ca/) (Pang et al. 2022), and metabolic pathway analysis was carried out with the pathway analysis tool of MetaboAnalyst 6.0 (Lu et al. 2023), using the hypergeometric test for pathway enrichment analysis and the out‐degree centrality for pathway topology analysis with the KEGG pathway library of *Arabidopsis thaliana* as reference (Avesani et al. 2023).

Manually curated annotation was carried out for annotated metabolic features with significant changes in abundance with LFC lower than −3 or higher than 3 and a *p* value of the *t*‐test lower than 0.05. Putative chemical names of each annotated metabolic feature found by the Compound Discoverer software (Version 3.3 SP2) were searched in the PubChem (https://pubchem.ncbi.nlm.nih.gov/), ChEBI, and KEGG databases to retrieve the exact mass, considering the ionization mode of detection, InChI code, InChIKey code, and spectral information (database reference spectra). Additional reference spectra were obtained in silico with InChI codes in the CFM‐ID 4.0 web server (Competitive Fragmentation Modeling for Metabolite Identification; https://cfmid.wishartlab.com) for each annotated metabolic feature. Database reference spectra and in silico reference spectra were visually compared with the experimental full scan MS spectra and ddMS2 fragmentation spectra of each annotated metabolic feature to select the most probable compound annotation, elemental formula, molecular ions, and database entry codes (annotated compound). Annotated compounds were then classified into nine putative chemical classes (benzenoids, carbohydrates and conjugates, carbonyl compounds, carboxylic acids and derivatives, indoles and derivatives, lipids and lipid‐like compounds, phenylpropanoids, terpenoids, and unknown) according to the manually curated annotation based on the classification obtained with the ClassyFire web‐based application (https://cfb.fiehnlab.ucdavis.edu/) by InChI code search (Djoumbou Feunang et al. 2016).

Authentic reference standards were used to validate compound annotation of β‐cyclocitral (Sigma‐Aldrich, Merck), β‐cyclocitric acid (in‐house production according to d'Alessandro et al. (2019)), geranyl pyrophosphate (Sigma‐Aldrich, Merck), and *trans*‐resveratrol (Sigma‐Aldrich, Merck). Authentic reference standards were analyzed at the concentration of 10 mg L^−1^ in methanol:water (80:20; v v^−1^) by UHPLC‐HESI‐Orbitrap‐MS analysis as described above. For compound identification, MS spectra, ddMS2 fragmentation spectra, and retention times of annotated compounds were compared with those of authentic reference standards.

### 2.9 Correlation Analysis of Transcriptomic and Metabolomic Data

Co‐expression network analysis of DETs was performed with a weighted gene co‐expression network analysis (WGCNA; v1.72.1) (Langfelder and Horvath 2008) using the Log_2_ (RPM + 1) values with a soft threshold power of 24 and a minimum of 15 transcripts per module (Vergata et al. 2023). WGCNA was also used to calculate the correlation of co‐expression modules with the profiles of annotated compounds, and relationships between the modules of DETs and metabolomic features were established by Pearson's correlation method (Pearson's coefficient ≥ 0.6 and *p* value ≤ 0.05).

## 3 Results

### 3.1 2‐Phenylethanol and β‐Cyclocitral Decreased Downy Mildew Severity in Grapevine Leaves and Modulated the Expression of Transcripts Related to Defense, Oxidative Stress, Secondary Metabolism, Signal Transduction, and Transcription

Treatments with 2‐phenylethanol and β‐cyclocitral decreased downy mildew severity on grapevine leaf disks with a disease reduction of 76.1% ± 2.6% and 82.4% ± 3.4%, respectively (Table 1). Transcriptomic and metabolomic analyses were carried out on leaf disks treated with water (control), 2‐phenylethanol, or β‐cyclocitral, inoculated with *P. viticola* ( *P. viticola* ‐inoculated) or treated with water (mock‐inoculated), and collected at 1 dpi and 6 dpi. A total of 607,661,079 paired‐end reads were obtained by RNA‐Seq analysis (Table S2) and were deposited at the Sequence Read Archive of the NCBI (https://www.ncbi.nlm. nih.gov/sra) under the BioProject number PRJNA871393. From 86 to 96% of filtered paired‐end reads aligned to the grapevine genome ( *V. vinifera* PN40024.v4 assembly), and 20,667 predicted transcripts resulted as active (RPM > 1 in at least two libraries; Table S3). MDS plot showed clustering of replicates and separation of samples according to the time point in the second dimension (Figure S2). The accuracy of RNA‐Seq results was validated by qPCR using seven genes (Table S1), and a good correlation (*R* ^2^ = 0.97) was obtained between expression levels assessed by RNA‐Seq and qPCR (Figure S3).

**TABLE 1:** Effects of 2‐phenylethanol and β‐cyclocitral against downy mildew severity.

Treatment^a^	Severity (%)^b^
Control	75.6 ± 3.6 a
2‐phenylethanol	18.0 ± 2.3 b
β‐cyclocitral	13.3 ± 4.7 b

^a^ Grapevine leaf disks were treated with water (Control), 2‐phenylethanol (20 mg L^−1^ in air volume in the dish chamber), or β‐cyclocitral (20 mg L^−1^ in air volume in the dish chamber).

^b^ Disks were inoculated with *Plasmopara viticola*, and downy mildew severity was assessed at 6 days post inoculation. Mean and standard error values of 20 replicates (dishes with six leaf disks each) are reported for each treatment. Different letters indicate significant differences among treatments according to the Kruskal‐Wallis test with Dunn's post hoc test (*p* ≤ 0.05).

A total of 4040 DETs were found by differential expression analysis of seven pairwise comparisons for each time point (Tables S4 and S5; LFC lower than −2 or higher than 2 and FDR lower than 0.05). In particular, β‐cyclocitral treatment caused a broad transcriptional response at 1 and 6 dpi in *P. viticola* ‐inoculated and mock‐inoculated samples, while 2‐phenylethanol treatment caused major transcriptional changes at 6 dpi (Figure S4A,B). DETs upregulated only in 2‐phenylethanol‐treated compared to control leaf disks were 48 at 1 dpi and 570 at 6 dpi in mock‐inoculated samples, 22 at 1 dpi and 16 at 6 dpi in *P. viticola* ‐inoculated samples (Figure S4C,D). DETs upregulated only in β‐cyclocitral‐treated compared to control leaf disks were 149 at 1 dpi and 107 at 6 dpi in mock‐inoculated samples, 324 at 1 dpi and 170 at 6 dpi in *P. viticola* ‐inoculated samples. Moreover, 46 and 293 DETs were downregulated only by 2‐phenylethanol treatment in mock‐inoculated samples, 111 and 66 DETs in *P. viticola* ‐inoculated samples at 1 dpi and 6 dpi, respectively (Figure S4E,F). DETs downregulated only in β‐cyclocitral‐treated compared to control leaf disks were 156 at 1 dpi and 63 at 6 dpi in mock‐inoculated samples, 852 at 1 dpi and 592 at 6 dpi in *P. viticola* ‐inoculated samples.

**FIGURE 1:**
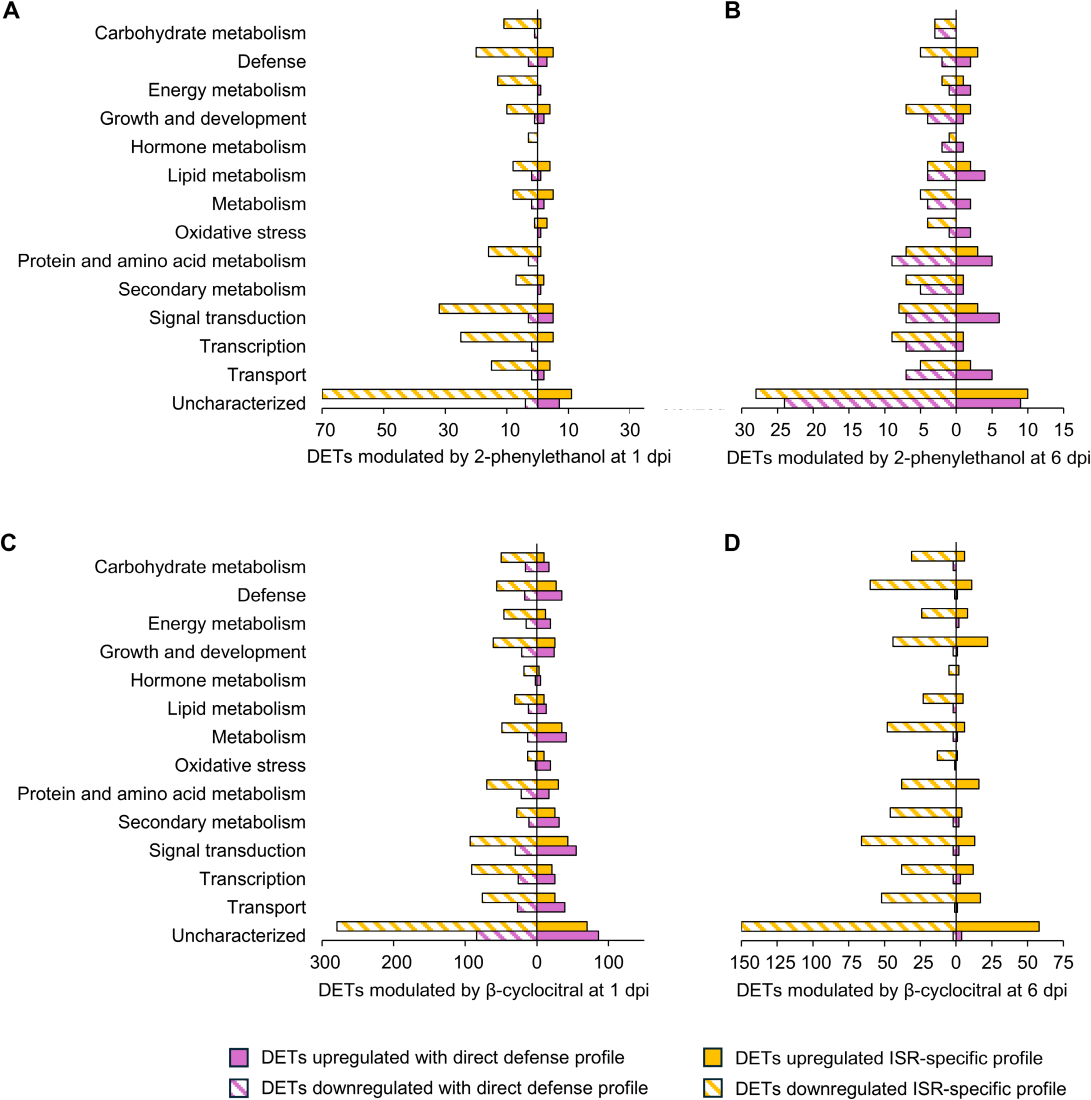
Functional annotation of differentially expressed transcripts (DETs). Upregulated (solid bars) and downregulated (stripped bars) DETs (Log_2_‐transformed fold change lower than −2 or higher than 2, and false discovery rate lower than 0.05) in grapevine leaf disks treated with water (Control), 2‐phenylethanol (A, B), or β‐cyclocitral (C, D), and inoculated with *Plasmopara viticola* ( *P. viticola* ‐inoculated) or water (Mock‐inoculated), were grouped in those with direct defense profile (purple; modulation in VOC‐treated compared to control leaf disks in both mock‐inoculated and *P. viticola* ‐inoculated samples) or ISR‐specific profile (yellow; modulation in VOC‐treated compared to control leaf disks only in *P. viticola* ‐inoculated samples) at one (A, C) and six (B, D) days post inoculation (dpi). Numbers of DETs are reported for each functional category, according to the manually curated annotation based on the protein description search and GO biological process annotations.

**FIGURE 2:**
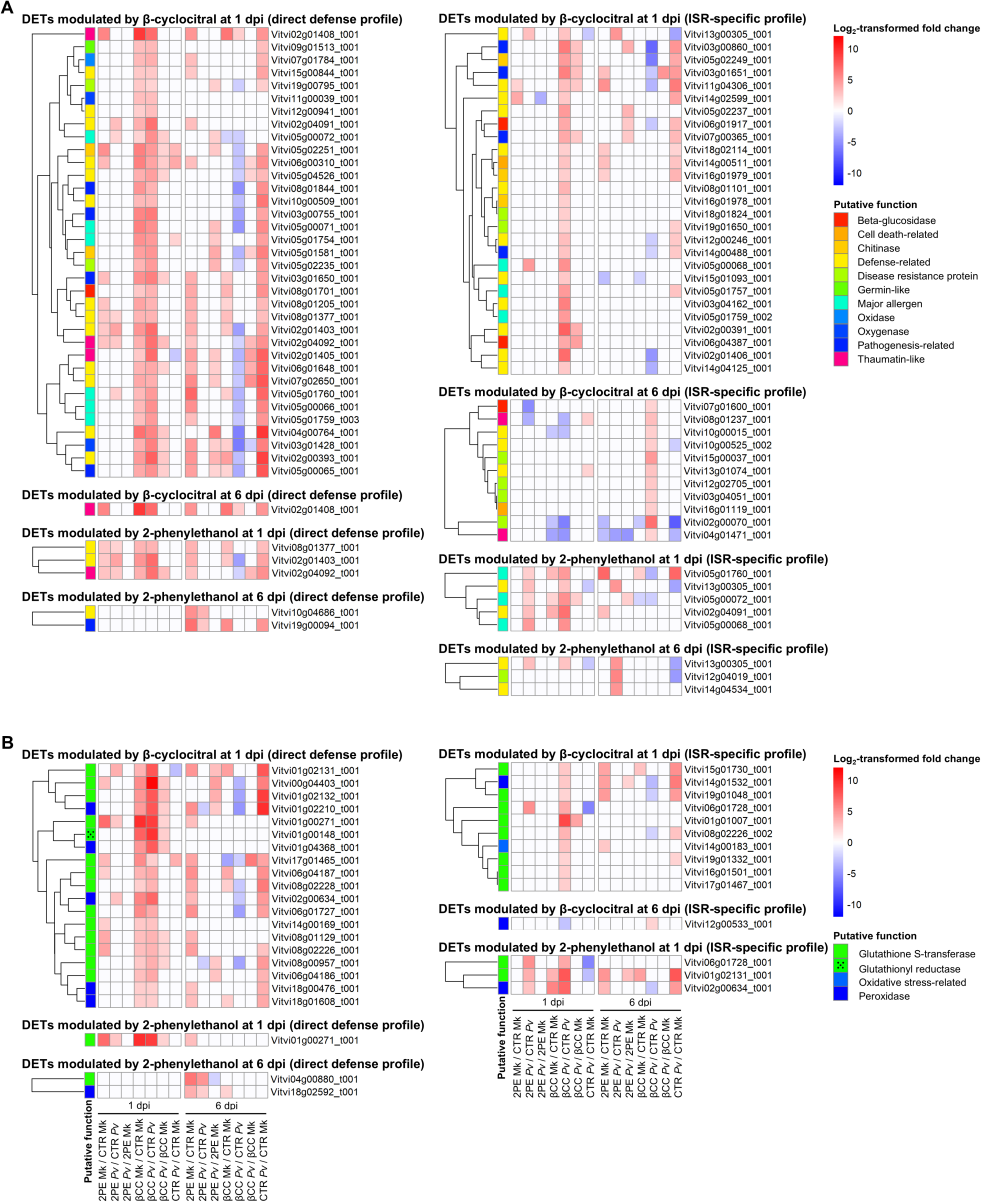
Heatmaps of differentially expressed transcripts (DETs) involved in defense (A) and oxidative stress (B). Log_2_‐transformed fold change values (color legend; LFC) of DETs (LFC lower than −2 or higher than 2 and false discovery rate lower than 0.05) are reported for pairwise comparisons of grapevine leaf disks treated with water (Control; CTR), 2‐phenylethanol (2PE), or β‐cyclocitral (βCC), inoculated with *Plasmopara viticola* (Pv) or water (Mk; mock‐inoculated) and collected at 1 or 6 days post inoculation (dpi). Putative functions (color legend) of DETs with direct defense profile (modulation in VOC‐treated compared to control leaf disks in mock‐inoculated and *P. viticola* ‐inoculated samples) or ISR‐specific profile (modulation in VOC‐treated compared to control leaf disks only in *P. viticola* ‐inoculated samples) were assigned according to the manually curated annotation based on the protein description search and GO biological process annotations.

**FIGURE 3:**
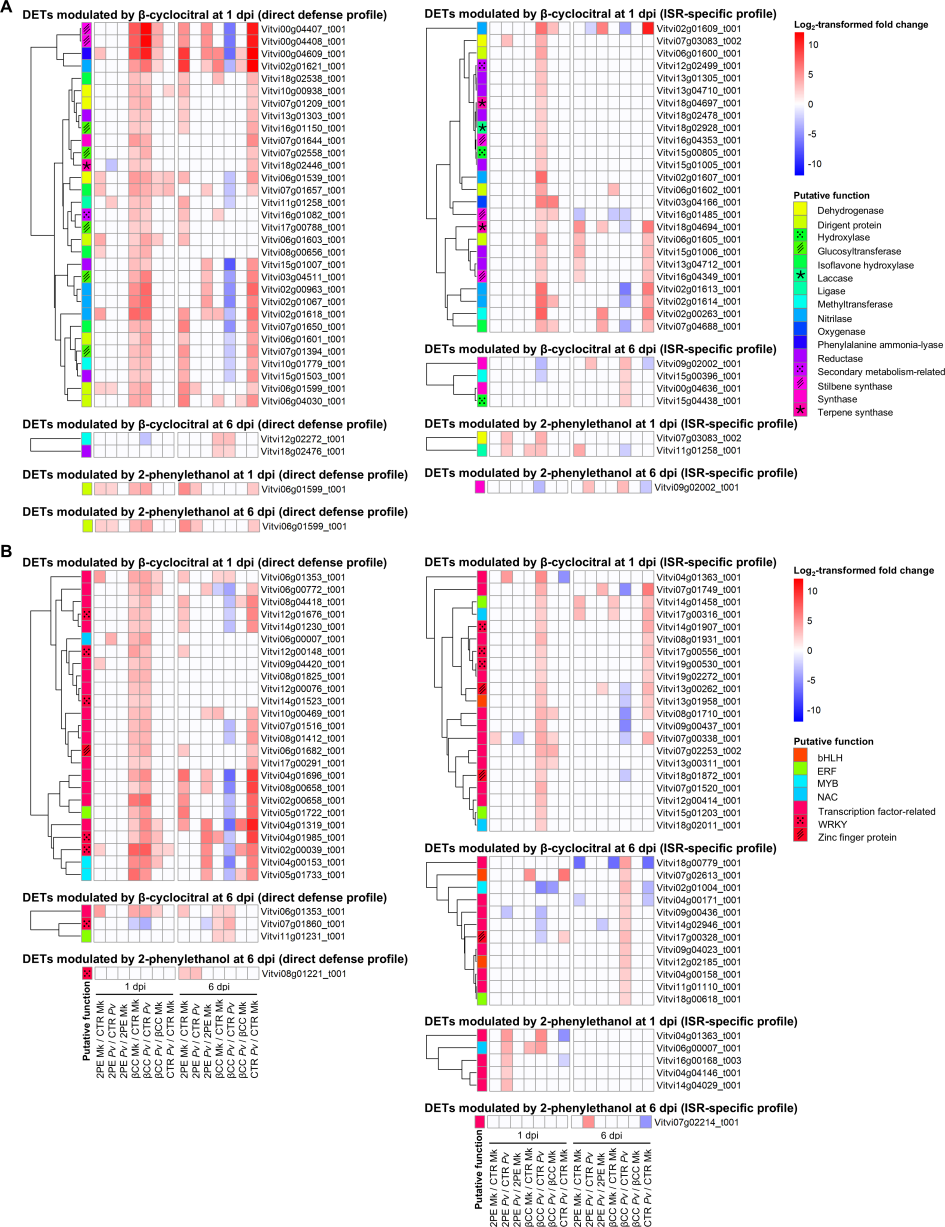
Heatmaps of differentially expressed transcripts (DETs) involved in secondary metabolism (A) and transcription (B). Log_2_‐transformed fold change values (color legend; LFC) of DETs (LFC lower than −2 or higher than 2 and false discovery rate lower than 0.05) are reported for pairwise comparisons of grapevine leaf disks treated with water (Control; CTR), 2‐phenylethanol (2PE), or β‐cyclocitral (βCC), inoculated with *Plasmopara viticola* (Pv) or water (Mk; mock‐inoculated) and collected at 1 or 6 days post inoculation (dpi). Putative functions (color legend) of DETs with direct defense profile (modulation in VOC‐treated compared to control leaf disks in mock‐inoculated and *P. viticola* ‐inoculated samples) or ISR‐specific profile (modulation in VOC‐treated compared to control leaf disks only in *P. viticola* ‐inoculated samples) were assigned according to the manually curated annotation based on the protein description search and GO biological process annotations.

Although DETs with unknown functions were found, upregulated and downregulated transcripts in 2‐phenylethanol‐treated samples with direct defense profile (modulation in VOC‐treated compared to control leaf disks in both mock‐inoculated and *P. viticola* ‐inoculated samples) were mainly associated with the functional categories of defense and signal transduction at 1 dpi (Figure 1A); protein and amino acid metabolism, signal transduction, and transport at 6 dpi (Figure 1B and Table S5). Moreover, DETs upregulated and downregulated by 2‐phenylethanol treatment with ISR‐specific profile (modulation in VOC‐treated compared to control leaf disks only in *P. viticola* ‐inoculated samples) were mainly associated with defense, signal transduction, and transcription at 1 dpi (Figure 1A); defense, protein and amino acid metabolism, signal transduction, and transcription at 6 dpi (Figure 1B and Table S5). Modulated transcripts in β‐cyclocitral‐treated samples with direct defense profile were mainly associated with defense, metabolism, secondary metabolism, signal transduction, and transport at 1 dpi (Figure 1C), and transcription at 6 dpi (Figure 1D and Table S5). Moreover, DETs modulated by β‐cyclocitral with an ISR‐specific profile were mainly associated with defense, metabolism, protein and amino acid metabolism, signal transduction, transcription, and transport at 1 dpi (Figure 1C); protein and amino acid metabolism, signal transduction, and transport at 6 dpi (Figure 1D and Table S5). In particular, DETs with direct defense and ISR‐specific profiles belonging to the defense category mainly encoded putative chitinases (5 transcripts), defense‐related proteins (30 transcripts), major allergen proteins (9 transcripts), PR proteins (9 transcripts), and thaumatin‐like proteins (5 transcripts; Figure 2A). DETs belonging to oxidative stress category mainly encoded putative glutathione S‐transferases (22 transcripts) and peroxidases (8 transcripts; Figure 2B), and those of secondary metabolism mainly encoded putative stilbene synthases (5 transcripts), terpene synthases (3 transcripts), and nitrilases (8 transcripts; Figure 3A). The functional category signal transduction included mainly DETs encoding kinases (8 transcripts), putative receptors (3 transcripts), receptor kinases (85 transcripts), and calmodulins (3 transcripts; Figure S5), and the functional category transcription included mainly DETs encoding putative bHLH (3 transcripts), ERF (5 transcripts), MYB (3 transcripts), NAC (3 transcripts), WRKY (10 transcripts) transcription factors, and zinc finger proteins (4 transcripts; Figure 3B). Moreover, most of the upregulated DETs with direct defense profile in 2‐phenylethanol and β‐cyclocitral leaf disks at 1 dpi were also upregulated in control leaf disks at 6 dpi with *P. viticola* , as possible VOC‐dependent pre‐activation of grapevine defense responses.

### 3.2 2‐Phenylethanol Affected the Abundance of Putative Carbohydrates and Conjugates, Putative Carboxylic Acids and Derivatives, and Putative Phenylpropanoids in Mock‐Inoculated and *Plasmopara viticola* ‐Inoculated Samples

A total of 13,475 metabolic features were detected in grapevine leaf disks by UHPLC‐HESI‐Orbitrap‐MS analysis (Table S6), and 4023 of them were annotated with the Compound Discoverer software (annotated metabolic features; Table S7). The PCA plot of detected metabolic features discriminated grapevine samples according to 2‐phenylethanol treatment and time point in the first and second components, respectively (Figure S6A–C). Annotated metabolic features with significant increases and decreases in abundance in leaf disks treated with 2‐phenylethanol were 467 and 173 at 1 dpi, and 650 and 531 at 6 dpi, respectively (LFC lower than −1 or higher than 1 and a *p* value of *t*‐test lower than 0.05; Table S8). In particular, annotated metabolic features with increased abundance in 2‐phenylethanol‐treated compared to control leaf disks were 134 and 27 in mock‐inoculated samples, 20 and 18 in *P. viticola* ‐inoculated samples, 290 and 193 in both inoculation conditions at 1 dpi (group 1) and 6 dpi (group 3), respectively (Figure S7A,B). Amino acid (phenylalanine, tyrosine, and tryptophan) biosynthesis, flavonoid biosynthesis, galactose, pentose, starch, and sucrose metabolism were found as enriched metabolic pathways (adjusted *p* value ≤ 0.05) of annotated metabolic features of group 1 and group 3 (Figure S7A,B; Table S9). Annotated metabolic features with increased abundance in *P. viticola* ‐inoculated compared to mock‐inoculated leaf disks were 23 at 1 dpi (group 2) and 412 at 6 dpi (group 4), and they were mainly related to galactose metabolism and flavonoid biosynthesis (Figure S7A,B; Table S9). Moreover, annotated metabolic features with decreased abundance in 2‐phenylethanol‐treated compared to control leaf disks were 133 at 1 dpi (group 5) and 436 at 6 dpi (group 7), and they revealed enriched metabolic pathways of galactose and tyrosine metabolism, isoquinoline alkaloid, phenylpropanoid (flavone, flavonol, and flavonoid), and terpenoid (sesquiterpenoid and triterpenoid) biosynthesis (Figure S7C,D; Table S9). Annotated metabolic features with decreased abundance in *P. viticola* ‐inoculated compared to mock‐inoculated leaf disks at 1 dpi (group 6) and 6 dpi (group 8) showed isoquinoline alkaloid and flavonoid biosynthesis, galactose, starch, and sucrose metabolism as enriched metabolic pathways (Figure S7C,D; Table S9).

**FIGURE 4:**
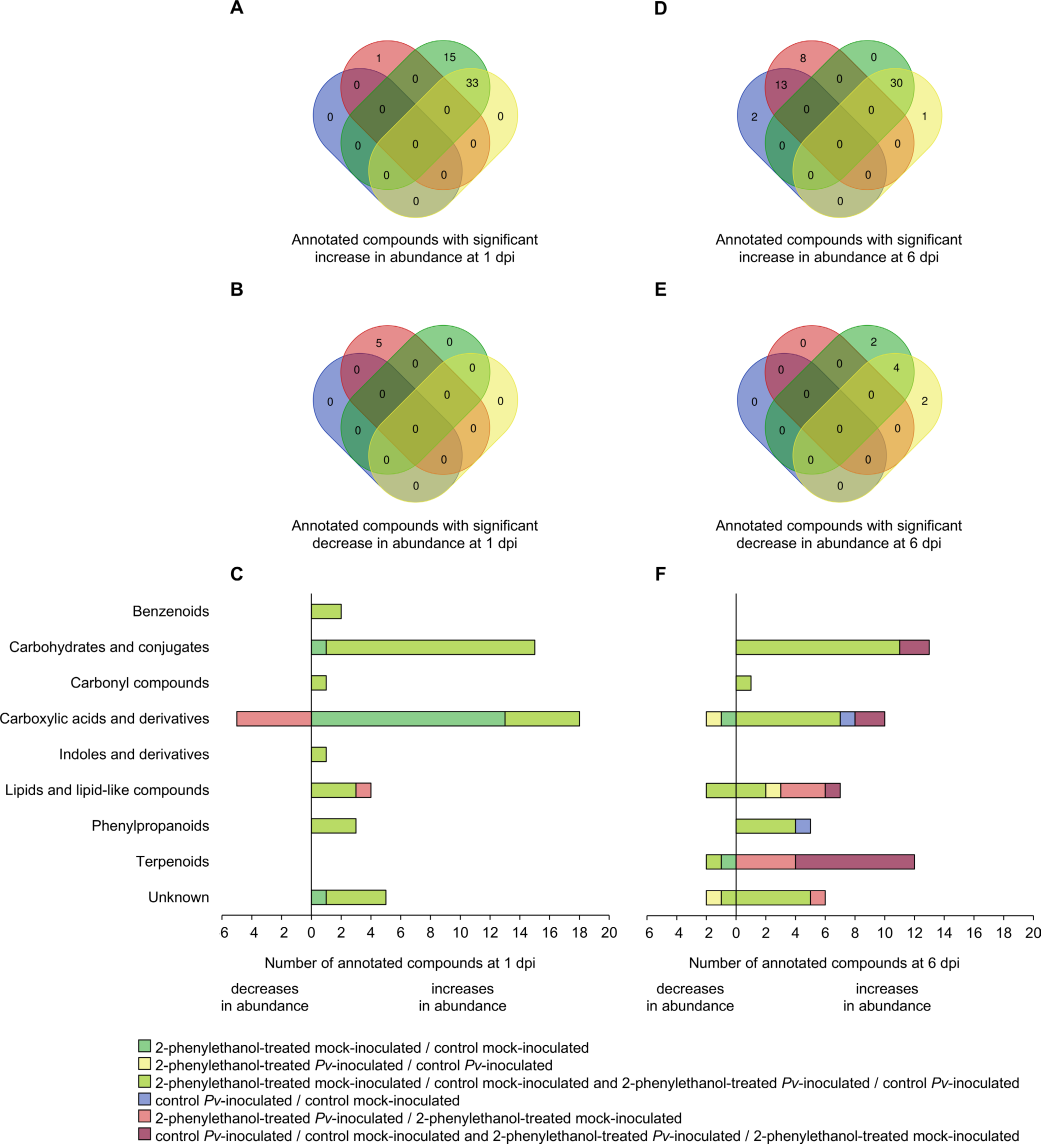
Annotated compounds with significant changes in abundance in leaf disks treated with 2‐phenylethanol. Venn diagrams summarize the distribution of annotated compounds (manually curated annotation) with significant increases (A, D) or decreases (B, E) in abundance (Log_2_‐transformed fold change lower than −3 or higher than 3, and *p* value of *t*‐test lower than 0.05) in the pairwise comparisons (color legend) of grapevine leaf disks treated with water (Control) or 2‐phenylethanol, inoculated with *Plasmopara viticola* (*Pv*‐inoculated) or water (Mock‐inoculated), and collected at 1 and 6 days post inoculation (dpi). Numbers of annotated compounds with significant increases or decreases in abundance in 2‐phenylethanol‐treated samples at 1 dpi (C) and 6 dpi (F) are reported for each chemical class (reported on the left of panel C), according to the pairwise comparisons specified by the color legend.

The manually curated annotation of metabolic features with significant changes in abundance in 2‐phenylethanol‐treated leaf disks (LFC lower than −3 or higher than 3 and a *p* value of *t*‐test lower than 0.05) allowed the classification of 116 annotated compounds (Figure 4A–F) into nine putative chemical classes (Figure 4C,F, Table S8). In particular, annotated compounds with increased abundance in 2‐phenylethanol‐treated compared to control leaf disks were 15 in mock‐inoculated samples, zero in *P. viticola* ‐inoculated samples, and 33 in both inoculation conditions at 1 dpi, and they mainly belonged to putative carbohydrates and conjugates, and putative carboxylic acids and derivatives (Figure 4A–C, Table S8). Moreover, one and five annotated compounds showed increases and decreases in abundance in *P. viticola* ‐inoculated compared to mock‐inoculated leaf disks in 2‐phenylethanol‐treated samples at 1 dpi, respectively (Figure 4A,B). At 6 dpi, the abundance of zero, one, and 30 annotated compounds increased in 2‐phenylethanol‐treated compared to control leaf disks in mock‐inoculated samples, in *P. viticola* ‐inoculated samples, and in both inoculation conditions, respectively, while the abundance of two, two, and four annotated compounds decreased in the respective comparisons (Figure 4D,E). Annotated compounds with increased abundance in 2‐phenylethanol‐treated samples at 6 dpi mainly belonged to putative carbohydrates and conjugates, putative carboxylic acids and derivatives, and putative phenylpropanoids (Figure 4F, and Table S8). Moreover, annotated compounds with decreased abundance in 2‐phenylethanol‐treated samples at 6 dpi mainly belonged to putative carboxylic acids and derivatives, putative lipids and lipid‐like compounds, and putative terpenoids (Figure 4F and Table S8). *Plasmopara viticola* inoculation increased the abundance of two annotated compounds in control samples, eight annotated compounds in 2‐phenylethanol‐treated samples, and 13 annotated compounds in both treatment conditions at 6 dpi (Figure 4D), and they mainly belonged to putative terpenoids and putative lipids and lipid‐like compounds (Figure 4F and Table S8).

### 3.3 β‐Cyclocitral Affected the Abundance of Putative Carboxylic Acids and Derivatives, and Putative Terpenoids in Mock‐Inoculated and *Plasmopara viticola* ‐Inoculated Samples

β‐cyclocitral treatment and time point discriminated grapevine samples in the first and second components of PCA of metabolic features detected by UHPLC‐HESI‐Orbitrap‐MS analysis, respectively (Figure S6D,E). Annotated metabolic features with significant increases and decreases in abundance in leaf disks treated with β‐cyclocitral were 682 and 413 in the pairwise comparisons at 1 dpi, 868 and 591 in the pairwise comparisons at 6 dpi, respectively (LFC lower than −1 or higher than 1 and a *p* value of *t*‐test lower than 0.05; Table S10). Annotated metabolic features with increased abundance in β‐cyclocitral‐treated compared to control leaf disks were 177 and 62 in mock‐inoculated samples, 25 and 33 in *P. viticola* ‐inoculated samples, 531 and 566 in both inoculation conditions at 1 dpi (group 9) and 6 dpi (group 11), respectively (Figure S8A,B). Galactose metabolism, glycolysis and gluconeogenesis, flavonoid biosynthesis, and monoterpenoid metabolism were found as enriched metabolic pathways (adjusted *p* value ≤ 0.05) of annotated metabolic features of group 9 and group 11 (Figure S8A,B; Table S11). Annotated metabolic features with increased abundance in *P. viticola* ‐inoculated compared to mock‐inoculated leaf disks were nine at 1 dpi (group 10) and 207 at 6 dpi (group 12), and they revealed enriched metabolic pathways (adjusted *p* value ≤ 0.05) of flavonoid biosynthesis (Figure S8A,B; Table S11). Moreover, annotated metabolic features with decreased abundance in β‐cyclocitral‐treated compared to control leaf disks were 375 at 1 dpi (group 13) and 559 at 6 dpi (group 15), and they showed flavonoid biosynthesis, amino acid, and galactose metabolism as enriched metabolic pathways (Figure S8C,D; Table S11). Annotated metabolic features with decreased abundance in *P. viticola* ‐inoculated compared to mock‐inoculated leaf disks at 1 dpi (group 14) and 6 dpi (group 16) were mainly related to carotenoid biosynthesis, glutathione metabolism, glycine, serine, and threonine metabolism (Figure S8C,D; Table S11).

**FIGURE 5:**
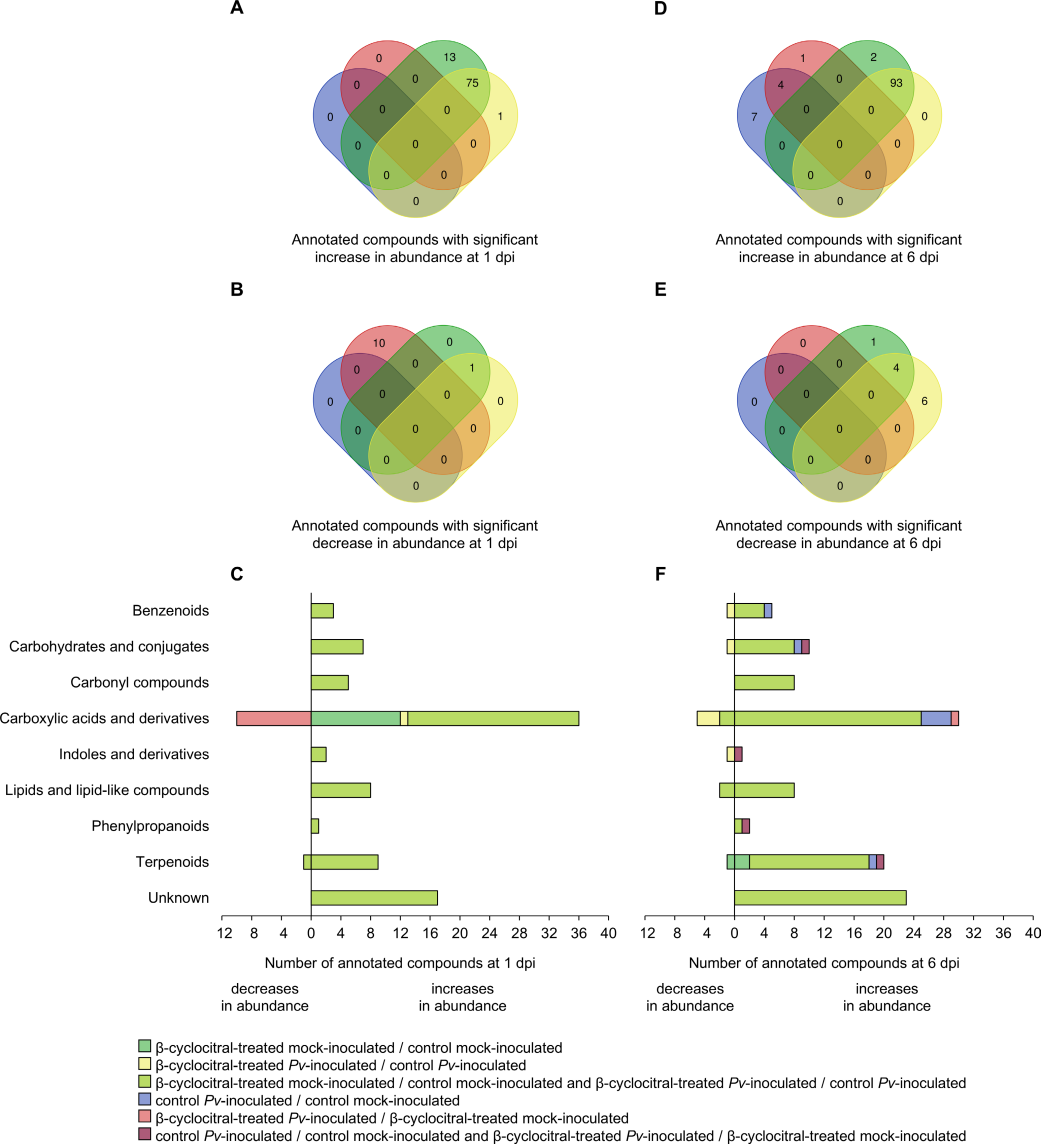
Annotated compounds with significant changes in abundance in leaf disks treated with β‐cyclocitral. Venn diagrams summarize the distribution of annotated compounds (manually curated annotation) with significant increases (A, D) or decreases (B, E) in abundance at 1 and 6 dpi (Log_2_‐transformed fold change lower than −3 or higher than 3, and *p* value of *t*‐test lower than 0.05) in the pairwise comparisons (color legend) of grapevine leaf disks treated with water (Control) or β‐cyclocitral, inoculated with *Plasmopara viticola* (*Pv*‐inoculated) or water (Mock‐inoculated), and collected at 1 and 6 days post inoculation (dpi). Numbers of annotated compounds with significant increases or decreases in abundance in β‐cyclocitral‐treated samples at 1 dpi (C) and 6 dpi (F) are reported for each chemical class (reported on the left of panel C), according to the pairwise comparisons specified by the color legend.

The manually curated annotation of metabolic features with significant changes in abundance in β‐cyclocitral‐treated leaf disks (LFC lower than −3 or higher than 3 and a *p* value of *t*‐test lower than 0.05) allowed the classification of 218 annotated compounds (Figure 5A–F) into nine putative chemical classes (Figure 5C,F, Table S10). Annotated compounds with increased abundance in β‐cyclocitral‐treated compared to control leaf disks at 1 dpi were 13 in mock‐inoculated samples, one in *P. viticola* ‐inoculated samples, and 75 in both inoculation conditions, respectively (Figure 5A,B), and they mainly belonged to putative carboxylic acids and derivatives and putative terpenoids (Figure 5C, Table S10). Moreover, 10 annotated compounds showed decreases in abundance in *P. viticola* ‐inoculated compared to mock‐inoculated leaf disks in β‐cyclocitral‐treated samples at 1 dpi (Figure 5B), and they belonged to putative carboxylic acids and derivatives (Figure 5C, Table S10). At 6 dpi, the abundance of two, zero, and 93 annotated compounds increased in β‐cyclocitral‐treated compared to control leaf disks in mock‐inoculated samples, in *P. viticola* ‐inoculated samples, and in both inoculation conditions, respectively, while the abundance of one, six, and four annotated compounds decreased in the respective comparisons (Figure 5D,E). Annotated compounds with increased abundance in β‐cyclocitral‐treated samples at 6 dpi mainly belonged to putative carboxylic acids and derivatives and putative terpenoids, while those with decreased abundance mainly belonged to putative carboxylic acids and derivatives (Figure 5F and Table S10). *Plasmopara viticola* inoculation increased the abundance of seven annotated compounds in control samples, one annotated compound in β‐cyclocitral‐treated samples, and four annotated compounds in both treatment conditions at 6 dpi (Figure 5D), and they mainly belonged to putative carboxylic acids and derivatives (Figure 5F and Table S10).

The analysis of authentic reference standards confirmed the identification of compounds affected by 2‐phenylethanol and/or β‐cyclocitral treatment, as indicated by the comparison of MS spectra and ddMS2 fragmentation spectra of geranyl pyrophosphate, *trans*‐resveratrol, β‐cyclocitral, and β‐cyclocitric acid (Figure S9).

### 3.4 Correlations of Transcriptomic and Metabolomic Data

Co‐expression network of 4040 DETs highlighted eight modules (Figure S10, Table S5), and they were correlated with 198 annotated compounds with significant changes in abundance and assigned to a putative chemical class (Table S12). In particular, putative carbohydrates and conjugates (6‐acetyl‐D‐glucose, fructosamine, syringin, and penicitriketo) were positively correlated (correlation higher than 0.6 and *p* value lower than 0.05) with module 3 and module 7, which included transcripts related mainly to defense, protein and amino acid metabolism, signal transduction, transcription, and transport. Likewise, putative carboxylic acids and derivatives [(*4Z*, *7Z*)‐4,7‐decadienyl sulfate, 5‐aminopentanoate, arginine, varioxiranediol, and glutamylcysteine] were positively correlated mainly with module 3, module 5, and module 7, which included transcripts related to defense (e.g., chitinases and defense‐related proteins), signal transduction (e.g., calcium‐binding proteins, calmodulins, and receptor kinases), and transcription (e.g., ERF and MYB genes). Moreover, putative terpenoids were positively correlated with module 1 (verbenone or pinocarvone, and isopimara‐8,15‐dien‐19‐ol), module 3 (oleanolic acid), module 5 [24,25‐epoxy‐3‐oxolanosta‐7,9(11)‐diene and ganoderiol], and module 6 (gibberellin) that included carotene isomerase (Vitvi02g00235_t001), geraniol hydroxylase (Vitvi02g01537_t001), and several transcription factor genes. Putative phenylpropanoids (vitexin‐2‐*O*‐rhamnoside and trans‐resveratrol) were positively correlated with module 7, which included three transcripts possibly involved in phenylpropanoid glycosylation (Vitvi06g04208_t001, Vitvi06g04210_t001, and Vitvi06g04211_t001) and signal transduction, such as receptor kinases (e.g., Vitvi10g04382_t001, and Vitvi17g00854_t001) and phosphatases (e.g., Vitvi09g01282_t001).

## 4 Discussion

**FIGURE 6:**
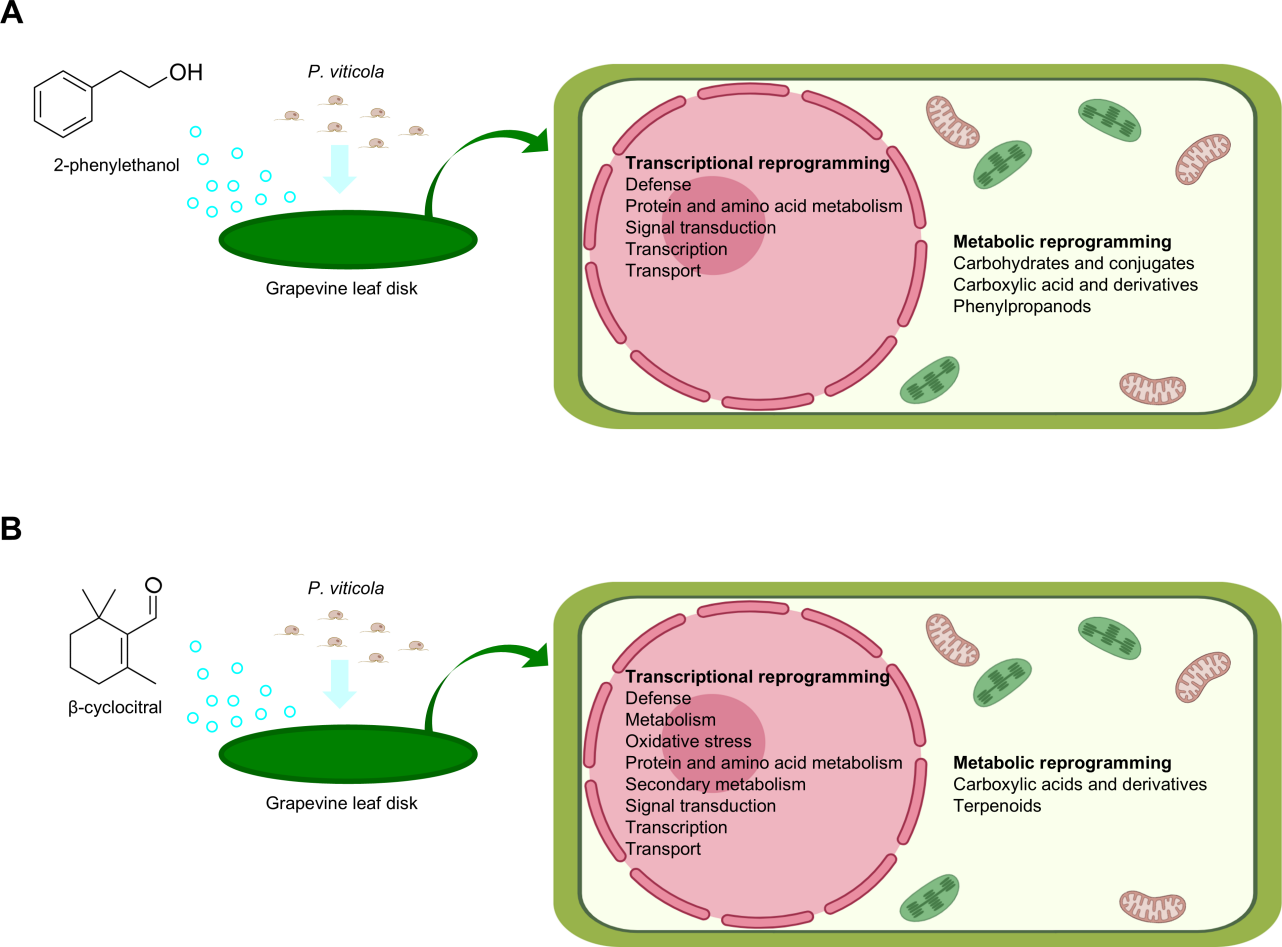
Schematic representation of the possible mode of action of volatile organic compounds against grapevine downy mildew. 2‐phenylethanol (A) and β‐cyclocitral (B) treatments cause transcriptional and metabolic responses in grapevine leaf disks. The main functional categories and chemical classes implicated in VOC‐dependent activation of plant defense mechanisms against downy mildew are summarized.

2‐phenylethanol and β‐cyclocitral are bioactive VOCs involved in plant–plant communication and plant resistance induction (Deshpande and Mitra 2023; Deshpande et al. 2021; Faizan et al. 2022; Kumar et al. 2024; Liu et al. 2016; Lv et al. 2015; Sun et al. 2021; Taniguchi et al. 2023). In grapevine, 2‐phenylethanol and β‐cyclocitral are emitted by downy mildew‐resistant genotypes (Chitarrini et al. 2020, 2017; Lazazzara et al. 2018; Ricciardi et al. 2021; Štambuk et al. 2023) and can decrease downy mildew severity on leaf disks of the susceptible genotype tested in this study (cultivar Pinot Noir). Moreover, 2‐phenylethanol and β‐cyclocitral did not affect the vitality of *P. viticola* sporangia (Lazazzara et al. 2018), suggesting no direct effects of both VOCs against this pathogen. The whole transcriptome and metabolome of grapevine leaf disks were affected by 2‐phenylethanol and β‐cyclocitral treatments, indicating the VOC‐dependent activation of plant defense mechanisms (Figure 6). In particular, leaf disk response was stronger to β‐cyclocitral compared to 2‐phenylethanol, in terms of the number of transcripts and metabolic features with changes in abundance. Transcriptomic data revealed that 2‐phenylethanol treatment mainly modulated the expression of transcripts related to defense, protein and amino acid metabolism, signal transduction, transcription, and transport, with direct defense and ISR‐specific profiles, indicating direct activation of defense‐related processes after VOC treatment and reinforcement of grapevine response upon *P. viticola* inoculation in 2‐phenylethanol‐treated leaf disks. Likewise, 2‐phenylethanol treatment can modulate the expression of genes related to cellular signaling, defense response, hormone‐mediated communication, phenylpropanoid biosynthesis, amino acid metabolism, and sugar metabolism in orange fruits (Liu et al. 2016). Moreover, 2‐phenylethanol treatment is known to upregulate the expression of genes related to defense mechanisms in tomato plants, such as endochitinase and lignin peroxidase genes (Kumar et al. 2024), indicating that 2‐phenylethanol can induce defense processes in receiver plants. In particular, genes related to defense, metabolism, signal transduction, secondary metabolism, and transcription are known to be upregulated by *P. viticola* inoculation in downy mildew‐resistant genotypes (Chitarrini et al. 2020; Liu et al. 2019; Polesani et al. 2010), suggesting that 2‐phenylethanol treatment can activate, in the susceptible grapevines, defense mechanisms that are commonly activated in downy mildew‐resistant genotypes. Moreover, annotated compounds with increased abundance in 2‐phenylethanol‐treated compared to control leaf disks mainly belonged to putative carbohydrates and conjugates, putative carboxylic acids and derivatives, and putative phenylpropanoids at 1 and/or 6 dpi. Likewise, carbohydrates and carboxylic acids were previously found in downy mildew‐resistant genotypes upon *P. viticola* inoculation (Chitarrini et al. 2020; Ciubotaru et al. 2021) and in grapevine berries upon *B. cinerea* inoculation toward increased synthesis of secondary metabolites involved in plant defense (Agudelo‐Romero et al. 2015). For example, some amino acids (e.g., arginine) and carboxylic acids (e.g., glutathione) found in 2‐phenylethanol‐treated samples are known to accumulate in grapevine leaves upon *B. cinerea* inoculation and to contribute to grapevine defense (Jia et al. 2022). Likewise, some phenylpropanoids (e.g., aromadendrin, p‐coumaryl alcohol 4‐O‐glucoside, and vitexin‐2‐O‐rhamnoside) showed increased abundance in response to 2‐phenylethanol, and this class of compounds is known to be synthesized in defense response against *P. viticola* (Ali et al. 2012; Chitarrini et al. 2017; Ciubotaru et al. 2021; Malacarne et al. 2011). In particular, aromadendrin previously showed inhibitory activities against phytopathogens ( *P. syringae* pv. *tomato* and *Xanthomonas* spp.) (Kotan et al. 2014), and *p*‐coumaryl alcohol was previously associated with the lignification of Arabidopsis leaves in response to *P. syringae* pv. *tomato* DC3000 infection (Lee et al. 2019), corroborating that 2‐phenylethanol treatment can stimulate the accumulation of defense‐related compounds in grapevine leaf disks.

β‐cyclocitral treatment modulated the expression of transcripts mainly related to six functional categories (defense, metabolism, secondary metabolism, signal transduction, transcription, and transport) with a direct defense profile, and six functional categories (defense, metabolism, protein and amino acid metabolism, signal transduction, transcription, and transport) with an ISR‐specific profile. At 1 dpi, β‐cyclocitral upregulated the expression of eight and 13 glutathione S‐transferase genes with ISR‐specific and direct defense profiles, respectively, indicating the activation of ROS detoxification mechanisms. Likewise, β‐cyclocitral is known to trigger stress tolerance by modulating the expression of genes encoding transcription regulators and ROS detoxifying enzymes in plants (Faizan et al. 2022). For example, β‐cyclocitral can stimulate light acclimation by decreasing ROS production and upregulating the expression of glutathione S‐transferase genes in Arabidopsis (Lv et al. 2015). β‐cyclocitral can maintain the antioxidant system of quinoa seedlings, including enzymatic activities (e.g., ascorbate peroxidase, catalase, peroxidase, and superoxide dismutase) and non‐enzymatic antioxidants (e.g., reduced glutathione and ascorbic acid) (Sun et al. 2021). Moreover, β‐cyclocitral treatment can induce transcriptional reprogramming in rice (Taniguchi et al. 2023) and tomato (Deshpande et al. 2021), with the upregulation of genes related to abiotic and biotic stress responses, such as genes encoding peroxidase, calcium‐binding proteins, heat shock proteins, and transcription factors. Consistency of functional categories upregulated by β‐cyclocitral treatment (e.g., defense, metabolism, signal transduction, and transcription) with those upregulated by *P. viticola* inoculation in downy mildew‐resistant genotypes (Chitarrini et al. 2020; Liu et al. 2019; Polesani et al. 2010) suggested that this VOC can activate downy mildew resistance‐related pathways in susceptible grapevines. Moreover, annotated compounds with increased abundance in β‐cyclocitral‐treated compared to control leaf disks mainly belonged to putative carboxylic acids and derivatives (e.g., 3‐dehydroquinic acid) and putative terpenoids (e.g., 4‐thujanol, camphor, geranyl pyrophosphate, and perillaldehyde). For example, 3‐dehydroquinic acid is a precursor of the shikimate pathway, responsible for producing secondary metabolites implicated in plant defense responses (Maeda and Dudareva 2012). Moreover, some terpenoids found in β‐cyclocitral‐treated samples are known to display inhibitory activities against phytopathogens, such as 4‐thujanol (also known as sabinene hydrate) against *Pseudocercospora vitis* (Rozwalka et al. 2020), camphor against *Fusarium* spp. (Gazdağlı et al. 2018; Kong et al. 2022), perillaldehyde against *Aspergillus niger* (Tian et al. 2015), *B. cinerea* (Wang et al. 2023), and *Ceratocystis fimbriata* (Tian et al. 2019), indicating that β‐cyclocitral treatment can stimulate the accumulation of defense‐related compounds in grapevine leaf disks. Likewise, β‐cyclocitral treatment caused metabolic changes in tomato leaves and increased the abundance of compounds associated with plant growth and defense, such as those related to amino acid (e.g., aspartate, glutamate, homoserine, leucine, *o*‐acetylserine, threonine, tryptophan tyramine, and valine), phenylpropanoid (e.g., ferulic acid, coumaric acid, isoferulic acid, rutin, quinic acid, and shikimate), carbohydrate (e.g., galactose, glucose, and mannose), carboxylic acid (e.g., citric acid, fumaric acid, and salicylic acid), and lipid (e.g., α‐tomatine) metabolism (Deshpande and Mitra 2023), indicating that β‐cyclocitral can induce resistance‐related pathways.

Correlation analysis between transcriptomic and metabolomic data revealed that putative phenylpropanoids positively correlated with transcripts encoding leucine‐rich repeat receptor kinases and UDP‐glycosyltransferases that were previously associated with grapevine resistance against downy mildew (Fan et al. 2015; Liu et al. 2019). Moreover, putative carboxylic acids and derivatives correlated with transcripts encoding signal transduction proteins, transcription factors, and defense‐related proteins, such as protein P21 (also classified as osmotin) (Faillace et al. 2021), possibly involved in grapevine defense against pathogens (Monteiro et al. 2003). Likewise, arginine and its by‐product (5‐aminopentanoate) positively correlated with transcripts related to defense, signal transduction, and transcription, suggesting a contribution to grapevine defense reactions against downy mildew. However, a large fraction of unknown genes, uncharacterized compounds, and poorly annotated metabolic features was found in our study, indicating that further molecular and physiological studies are required to identify the functional roles of compounds affected by 2‐phenylethanol and β‐cyclocitral treatments.

Grapevine response to *P. viticola* revealed the reprogramming of two pathways (galactose metabolism and flavonoid biosynthesis) and six pathways (amino acid metabolism, carotenoid biosynthesis, glutathione metabolism, isoquinoline alkaloid biosynthesis, galactose metabolism, and starch/sucrose metabolism) with increases and decreases in abundance, respectively. Annotated compounds with increased abundance in *P. viticola* ‐inoculated compared to mock‐inoculated samples belonged to benzenoids, carbohydrates and conjugates, carboxylic acids and derivatives, indoles and derivatives, lipids and lipid‐like compounds, phenylpropanoids, and terpenoids, suggesting the activation of multiple defense pathways. In particular, *trans*‐resveratrol showed increased abundance in *P. viticola* ‐inoculated compared to mock‐inoculated samples treated with 2‐phenylethanol and β‐cyclocitral, and phenylpropanoids are known to be synthesized during grapevine response against *P. viticola* with specific profiles in downy mildew‐susceptible and resistant genotypes (Ali et al. 2012; Ciubotaru et al. 2021; Malacarne et al. 2011).

VOCs have great potential against plant pathogens, but the nature of these compounds makes them sensitive to evaporation (Sharifi et al. 2018). In our experiments, VOC contact with the plant tissue was ensured by incubation within the dish chamber, and this leaf disk assay is frequently used to analyze resistance induction mechanisms and to assess metabolic responses under controlled conditions (Adrian et al. 2017; Avesani et al. 2023). However, spray treatments and air volume applications on whole plants are required to validate the efficacy of 2‐phenylethanol and β‐cyclocitral against downy mildew and to investigate the VOC‐dependent activation of grapevine resistance mechanisms in planta. VOC applications have been proposed as sustainable strategies to limit phytopathogen infection and reduce the use of chemical fungicides on crops (Sharifi and Ryu 2018, 2021). Our results indicated that 2‐phenylethanol and β‐cyclocitral are promising molecules that could be used against grapevine downy mildew. Their mode of action relies mainly on plant resistance induction, suggesting that preventive applications are required to activate grapevine defenses efficiently. However, further studies on the application dosage, application timing, and product formulation are required to optimize VOC‐based strategies for grapevine protection under field conditions.

## 5 Conclusions

2‐phenylethanol and β‐cyclocitral decreased downy mildew severity in grapevine leaf disks, causing a complex reprogramming of the leaf transcriptome and metabolome. 2‐phenylethanol and β‐cyclocitral treatments upregulated the expression of transcripts related to defense response (e.g., chitinases, defense‐related proteins, PR proteins, and thaumatin‐like proteins), oxidative stress (e.g., glutathione S‐transferases and peroxidases), secondary metabolism (e.g., nitrilases, stilbene synthases, and terpene synthases), signal transduction (e.g., kinases, receptor kinases, and calmodulins), and transcription (e.g., bHLH, ERF, MYB, NAC, and WRKY transcription factors), possibly involved in defense mechanisms against *P. viticola* . Moreover, 2‐phenylethanol and β‐cyclocitral treatments increased the abundance of putative carbohydrates, carboxylic acids, phenylpropanoids, terpenoids, and derivatives, including molecules possibly associated with plant defense, such as 3‐dehydroquinic acid, 4‐thujanol, aromadendrin, camphor, *p*‐coumaryl alcohol, and perillaldehyde.

## Author Contributions

S.A. and V.L. carried out the experiments and qPCR analyses. M.B. and V.L. carried out the bioinformatic analyses of transcriptomic data and annotation of differentially expressed transcripts. S.A. carried out the metabolomic analysis and metabolite annotations. S.A., V.L., M.B., P.R., M.O., and M.P. contributed to data interpretation. M.P. and P.R. conceived the study, designed the experiment, and coordinated all research activities. S.A., V.L., and M.P. wrote the manuscript. All the authors revised and approved the final manuscript.

## Conflicts of Interest

The authors declare no conflicts of interest.

## Supporting information


**Figure S1.** Untargeted metabolomics workflow of Compound Discoverer. Grapevine leaf disk samples were analyzed using ultra‐high pressure liquid chromatography—heated electrospray ionization—Orbitrap mass spectrometry analysis. Full scan MS data and ddMS2 fragmentation data were processed using an untargeted metabolomics workflow (Untargeted Metabolomics with Statistics Detect Unknown with ID using Online Database and mzLogic) on Compound Discoverer. The nodular workflow involved spectrum selection (Select Spectra), chromatographic alignment (Align Retention Times), metabolic feature detection (Detect Compounds, Group Compounds, Fill Gaps), peak area normalization according to quality control samples (Apply SERRF QC Correction, Mark Background Compounds, Normalize Areas), chemical annotation of metabolic features (Assign Compound Annotations, Predict Compositions, Search ChemSpider, Search mzCloud nodes, Search Mass Lists, Apply mzLogic node and Search Neutral Losses). Metabolic features with significant changes in abundances were selected, imposing a Log2‐transformed fold change lower than −1 or higher than 1 and a *p* value of *t*‐test lower than 0.05 in seven pairwise comparisons for each time point (Differential Analysis).
**Figure S2.** Multi‐dimensional scaling (MDS) plot of transcriptomic data. Grapevine leaf disks were treated with water (Control; blue), 2‐phenylethanol (2PE‐treated; red), or β‐cyclocitral (βCC‐treated; green), inoculated with Plasmopara viticola (P. viticola‐inoculated; solid symbols) or water (Mock‐ inoculated). Three replicates (pool of ten leaf disks each) for each treatment were collected at one and six days post inoculation (dpi) and subjected to RNA‐Seq analysis. First and second dimension (A) or first and third dimension (B) of MDS plot were obtained with the EdgeR v3.38.4 tool on normalized counts of 20,667 active transcripts detected in grapevine samples 4.
**Figure S3.** RNA‐Seq data validation by qPCR. Heatmaps of Log2‐transformed fold change (LFC) values assessed by RNA‐Seq and qPCR are reported according to the color scale legend for transcript encoding chitinase 3 (CHIT‐3), hypersensitive response‐related gene (HSR), lipoxygenase 9 (LOX‐9), osmotin 2 (OSM‐2), pathogenesis‐related (PR) protein 2 (PR‐2) and 4 (PR‐4), and stilbene synthase gene (STS) in the pairwise comparisons of grapevine leaf disks treated with water (Control; CTR), 2‐phenylethanol (2PE), or β‐cyclocitral (βCC), inoculated with Plasmopara viticola (Pv) or water (Mock‐inoculated; Mk) and collected at one or six days post inoculation (dpi) (A). A scatter plot of LFC values of RNA‐Seq and qPCR analyses is reported with the regression line equation and the R2 coefficient value (B).
**Figure S4.** Differential expression analysis results. Numbers of upregulated (red bars) and downregulated (blue bars) differentially expressed transcripts (DETs; log2‐transformed fold change lower than −2 or higher than 2, and false discovery rate lower than 0.05) are reported for each pairwise comparison of grapevine leaf disks treated with water (Control; CTR), 2‐phenylethanol (2PE), or β‐cyclocitral (βCC), inoculated with Plasmopara viticola (P. viticola‐inoculated; Pv) or water (Mock‐inoculated; Mk), and collected at one (A) and six (B) days post inoculation (dpi). Venn diagrams summarize the distribution of upregulated (C, D) and downregulated (E, F) DETs at 1 dpi (C, E) and 6 dpi (D, F) in the pairwise comparisons specified by the color legend.
**Figure S5.** Heatmaps of differentially expressed transcripts (DETs) involved in signal transduction. Log2‐transformed fold change values (color legend; LFC) of DETs (LFC lower than −2 or higher than 2 and false discovery rate lower than 0.05) are reported for pairwise comparisons of grapevine leaf disks treated with water (Control; CTR), 2‐phenylethanol (2PE), or β‐cyclocitral (βCC), inoculated with Plasmopara viticola (Pv) or water (Mk; mock‐inoculated) and collected at one or six days post inoculation (dpi). Putative functions (color legend) of DETs with direct defense profile (modulation 7 in VOC‐treated compared to control leaf disks in mock‐inoculated and P. viticola‐inoculated samples) or ISR‐specific profile (modulation in VOC‐treated compared to control leaf disks only in P. viticola‐ inoculated samples) were assigned according to the manually curated annotation based on the protein description search and GO biological process annotations.
**Figure S6.** Principal component analysis (PCA) of metabolomic data. Grapevine leaf disks were treated with water (Control; blue), 2‐phenylethanol (red), or β‐cyclocitral (green), inoculated with Plasmopara viticola (P. viticola‐inoculated; solid symbols) or water (Mock‐inoculated; open symbols). Six replicates (pool of ten leaf disks each) for each treatment were collected at one (circles) and six (triangles) days post inoculation (dpi) and subjected to ultra high pressure liquid chromatography—heated electrospray ionization—Orbitrap mass spectrometry (UHPLC‐HESI‐Orbitrap‐MS) analysis. PCA was obtained with MetaboAnalyst on 13,475 metabolic features detected in grapevine samples. Quality control samples (gray squares) were obtained from the homogenization of equal aliquots of each sample.
**Figure S7.** Metabolic pathway analysis of annotated metabolic features with significant changes in abundances in leaf disks treated with 2‐phenylethanol. Venn diagrams summarize the distribution of annotated metabolic features with significant increases (A, B) and decreases (C, D) in abundance (Log2‐transformed fold change lower than −1 or higher than 1, and a *p* value of *t*‐test lower than 0.05) in the pairwise comparisons (color legend) of grapevine leaf disks treated with water (Control) or 2‐phenylethanol, inoculated with Plasmopara viticola (Pv‐inoculated) or water (Mock‐inoculated), and collected at one (A, C) and six (B, D) days post inoculation (dpi). Metabolic pathway analysis was carried out using the pathway analysis tool of MetaboAnalyst based on the Kyoto Encyclopedia of Genes and Genomes (KEGG) data of annotated metabolic features with increases (groups 1, 2, 3, and 4) or decreases (groups 5, 6, 7, and 8) in abundance reported in the Venn diagrams and affected mainly by 2‐phenylethanol treatment (groups 1, 3, 5, and 7) or P. viticola inoculation (groups 2, 4, 6, and 8). Pathways are arranged according to *p* values (y‐axis) of pathway enrichment analysis and pathway impact values (x‐axis) of pathway topology analysis. The node color and size of each pathway are determined by the *p* value (red color with the lowest *p* value) and impact value (diameter proportional to the impact), respectively. Enriched metabolic pathways (adjusted *p* value ≤ 0.05) are reported for each chart.
**Figure S8.** Metabolic pathway analysis of annotated metabolic features with significant changes in abundances in leaf disks treated with β‐cyclocitral. Venn diagrams summarize the distribution of annotated metabolic features with significant increases (A, B) and decreases (C, D) in abundance (Log2‐transformed fold change lower than −1 or higher than 1, and a *p* value of *t*‐test lower than 0.05) in the pairwise comparisons (color legend) of grapevine leaf disks treated with water (Control) or β‐cyclocitral, inoculated with Plasmopara viticola (Pv‐inoculated) or water (Mock‐inoculated), and collected at one (A, C) and six (B, D) days post inoculation (dpi). Metabolic pathway analysis was carried out using the pathway analysis tool of MetaboAnalyst based on the Kyoto Encyclopedia of Genes and Genomes (KEGG) data of annotated metabolic features with increases (groups 9, 10, 11, and 12) or decreases (groups 13, 14, 15, and 16) in abundance reported in the Venn diagrams and affected mainly by β‐cyclocitral treatment (groups 9, 11, 13, 15) or P. viticola inoculation (groups 10, 12, 14, 16). Pathways are arranged according to *p* values (y‐axis) of pathway enrichment analysis and pathway impact values (x‐axis) of pathway topology analysis. The node color and size of each pathway are determined by the *p* value (red color with the lowest *p* value) and impact value (diameter proportional to the impact), respectively. Enriched metabolic pathways (adjusted *p* value ≤ 0.05) are reported for each chart.
**Figure S9.** Comparison of the experimental mass spectra of annotated compounds found in grapevine leaf samples with the experimental mass spectra of the corresponding authentic reference standards: β‐cyclocitral (A), β‐cyclocitric acid (B), geranyl pyrophosphate (C), and trans‐resveratrol (D). The comparison of full scan mass (MS) spectra and data‐dependent tandem mass spectrometry (ddMS2) fragmentation spectra, retention time, and heated electrospray ionization (HESI) mode is reported for each compound.
**Figure S10.** Co‐expression network analysis of differentially expressed transcripts (DETs). Weighted co‐expression network analysis (WGCNA) of DETs was carried out using the Log2 (RPM+1), and the height of the cluster dendrogram represents the dissimilarity between transcripts within the seven co‐expression modules. The correlation of co‐expression modules with the annotated compounds revealed relationships (Pearson’s coefficient ≥ 0.6 and *p* value ≤ 0.05) between the modules of DETs and the profiles of annotated compounds (Table S12).

**Table S1.** Primer sequences of grapevine genes analyzed by quantitative real‐time PCR. Gene name (column A), gene abbreviation (column B), accession numbers in the National Center for Biotechnology Information (NCBI) database (http://www.ncbi.nlm.nih.gov; column C), and transcript ID (column D) of the grapevine genome (PN40024.v4.1; https://integrape.eu/resources/genes‐genomes/genome‐accessions) are reported for each gene. Sequences of the forward primer (column E) and reverse primer (column F) are indicated for each gene with the respective reference (column G).
**Table S2.** RNA‐Seq data elaboration statistics. RNA‐Seq data of grapevine leaf disk samples treated with water (Control), 2‐phenylethanol (2PE), or β‐cyclocitral (βCC), inoculated with Plasmopara viticola (Pv‐inoculated) or water (mock‐ inoculated), and collected in triplicate (named rep 1, rep 2, and rep 3) at one and six days post inoculation (dpi). The number of paired‐end raw reads (column E), the number and the percentage of filtered paired‐end reads after quality filtering (filtered read pairs; columns F and G), and the number and the percentage of paired‐end reads aligned to the grapevine reference genome (V. vinifera PN40024.v4; mapped read pairs; columns H and I) are reported for each sample.
**Table S3.** Expression levels of grapevine transcripts. For each grapevine transcript (V. vinifera PN40024.v4), expression levels are reported as fragments per kilobase million (RPM) for each sample of grapevine leaf disks treated with water (Control; CTR), 2‐phenylethanol (2PE), or β‐cyclocitral (βCC), inoculated with Plasmopara viticola (P. viticola‐inoculated; Pv) or water (Mock‐inoculated; Mk), and collected in triplicate (named rep 1, rep 2, and rep 3) at one and six days post inoculation (dpi; columns B‐AK).
**Table S4.** Differential expression analysis results. Grapevine leaf disks were treated with water (Control; CTR), 2‐phenylethanol (2PE), or β‐cyclocitral (βCC), inoculated with Plasmopara viticola (P. viticola‐inoculated; Pv) or water (Mock‐inoculated; Mk), and collected in triplicate at one and six days post inoculation (dpi). Differential expression analysis was carried out on active transcripts (RPM > 1 in at least two libraries) with the likelihood ratio test for seven pairwise comparisons for each timepoint (columns A, B, and C) imposing a Log2‐ transformed fold change (LFC) lower than −2 or higher than 2 and a false discovery rate (FDR) lower than 0.05. The number of downregulated transcripts (Column D), upregulated transcript (Column E), and total modulated transcripts (Column F) are reported for each pairwise comparison.
**Table S5.** Expression levels and functional annotations of differentially expressed transcripts (DETs). Grapevine leaf disks were treated with water (Control; CTR), 2‐phenylethanol (2PE), or β‐cyclocitral (βCC), inoculated with Plasmopara viticola (P. viticola‐inoculated; Pv) or water (Mock‐inoculated; Mk), and collected at one and six days post inoculation (dpi). Differentially expressed transcripts (DETs) were obtained imposing a Log2‐transformed fold change (LFC) lower than −2 or higher than 2 and a false discovery rate (FDR) lower than 0.05 with the likelihood ratio test for seven pairwise comparisons for each timepoint. For each DET (V. vinifera PN40024.v4), LFC values and FDR values are reported for each pairwise comparison (columns B‐AC). DETs were grouped in transcript with direct defense profile (columns AD, AF, AH, AJ, AL, AN, AP, AR; upregulation or downregulation in the pairwise comparisons between 2‐phenylethanol‐ or β‐cyclocitral‐treated compared to control leaf disks in both mock‐inoculated and P. viticola‐inoculated samples at 1 dpi or 6 dpi) or ISR‐specific profile (columns AE, AG, AI, AK, AM, AO, AQ, AS; upregulation or downregulation in the pairwise comparisons between 2‐phenylethanol‐ or β‐cyclocitral‐treated compared to control leaf disks in P. viticola‐inoculated samples and not modulated in mock‐inoculated samples at 1 dpi or 6 dpi), and they were classified into 14 functional categories (columns AT), according to the manually curated annotation based on the protein descriptions (column AU) and Gene Ontology (GO) annotations (column AV) of grapevine genome annotation (Vitis vinifera PN40024.v4.1). Putative functions of DETs belonging to five functional categories possibly involved in grapevine resistance against downy mildew (defense, secondary metabolism, signal transduction, oxidative stress, and transcription) are reported in column AW. Co‐expression network analysis was carried out with a weighted gene co‐expression network analysis (WGCNA; v1.72.1) using the Log2 (RPM+1) values with a soft threshold power of 24 and a minimum of 15 transcripts per module, and it is indicated for each DET with the respective color (column AX) and code (column AY), are reported for each DET.
**Table S6.** Metabolic features detected by ultra high pressure liquid chromatography—heated electrospray ionization—Orbitrap mass spectrometry (UHPLC‐HESI‐Orbitrap‐MS) analysis. Grapevine leaf disks were treated with water (Control), 2‐phenylethanol, or β‐cyclocitral, inoculated with Plasmopara viticola (P. viticola‐inoculated) or water (Mock‐inoculated), and collected at one and six days post inoculation (dpi). Detected metabolic features (column A), specified by mean mass to charge ratio (m/z; column B) and mean retention time (rt) expressed as minutes (column C), were detected by UHPLC‐HESI‐Orbitrap‐MS analysis in negative heated‐electrospray ionization (HESI) mode (blue cells in column A) and positive HESI mode (red cells in column A). Peak area (abundance) is reported for six replicates (pool of ten leaf disks each) of control mock‐inoculated samples at 1 dpi (columns D–I), 2‐phenylethanol‐ 19 treated mock‐inoculated samples at 1 dpi (columns J‐O), β‐cyclocitral‐treated mock‐inoculated samples at 1 dpi (columns P‐U), control P. viticola‐inoculated samples at 1 dpi (columns V‐AA), 2‐phenylethanol‐treated P. viticola‐inoculated samples at 1 dpi (columns AB‐AG), β‐cyclocitral‐treated P. viticola‐inoculated samples at 1 dpi (columns AH‐AM), control mock‐inoculated samples at 6 dpi (columns AN‐AS), 2‐phenylethanol‐treated mock‐inoculated samples at 6 dpi (columns AT‐AY), β‐cyclocitral‐treated mock‐inoculated samples at 6 dpi (columns AZ‐BE), control P. viticola‐inoculated samples at 6 dpi (columns BF‐BK), 2‐phenylethanol‐treated P. viticola‐inoculated samples at 6 dpi (columns BL‐BQ), β‐cyclocitral‐treated P. viticola‐inoculated samples at 6 dpi (columns BR‐BW). Quality control (QC) samples were obtained from the homogenization of equal aliquots of each sample (columns BX‐CL). Metabolic features were annotated with Compound Discoverer and the most probable chemical name (column CM), elemental formula (column CN), annotation delta mass (column CO), calculated molecular weight (column CP), fragmentation information (column CQ), neutral losses (column CR), reference ion (column CS), number of Chem Spider results (column CT), number of mzCloud results (column CU) are reported for each feature. No chemical name and/or chemical formula was found for metabolic features with empty cells of columns CM, CN, and CO.
**Table S7.** Annotated metabolic features detected by ultra high pressure liquid chromatography ‐ heated electrospray ionization—Orbitrap mass spectrometry (UHPLC‐HESI‐Orbitrap‐MS) analysis. Grapevine leaf disks were treated with water (Control), 2‐phenylethanol or β‐cyclocitral, inoculated with Plasmopara viticola (P. viticola‐inoculated) or water (Mock‐inoculated), and collected at one and six days post inoculation (dpi). Metabolic features (column A), specified by mean mass to charge ratio (m/z; column B) and mean retention time (rt) expressed as minutes (column C), were detected by UHPLC‐HESI‐Orbitrap‐MS analysis in negative heated‐electrospray ionization (HESI) mode (blue cells in column A) and positive HESI mode (red cells in column A). Mean, standard error and coefficient of variation values of peak area (abundance) from six replicates (pool of ten leaf disks each) are reported for control mock‐inoculated samples at 1 dpi (columns D–F), 2‐phenylethanol‐treated mock‐inoculated samples at 1 dpi (columns G–I), β‐cyclocitral‐treated mock‐inoculated samples at 1 dpi (columns J–L), control P. viticola‐inoculated samples at 1 dpi (columns M–O), 2‐phenylethanol‐treated P. viticola‐inoculated samples at 1 dpi (columns P–R), β‐cyclocitral‐treated P. viticola‐inoculated samples at 1 dpi (columns S–U), control mock‐inoculated samples at 6 dpi (columns V‐X), 2‐phenylethanol‐treated mock‐ inoculated samples at 6 dpi (columns Y‐AA), β‐cyclocitral‐treated mock‐inoculated samples at 6 dpi (columns AB‐AD), control P. viticola‐inoculated samples at 6 dpi (columns AE‐AG), 2‐phenylethanol‐treated P. viticola‐inoculated samples at 6 dpi (columns AH‐AJ), β‐cyclocitral‐treated P. viticola‐inoculated samples at 6 dpi (columns AK‐AM). 20 Metabolic features with significant increases (UP) or decreases (DOWN) in abundances were selected with Compound Discoverer, imposing a Log2‐transformed fold change lower than −1 or higher than 1 and a *p* value of *t*‐test lower than 0.05 in at least one pairwise comparison, such as between control P. viticola‐inoculated and control mock‐inoculated samples at 1 dpi (columns AN‐AP), 2‐phenylethanol‐treated P. viticola‐inoculated and 2‐phenylethanol‐treated mock‐inoculated samples at 1 dpi (columns AQ‐AS), 2‐phenylethanol‐treated mock‐inoculated and control mock‐inoculated samples at 1 dpi (columns AT‐AV), 2‐phenylethanol‐treated P. viticola‐inoculated and control P. viticola‐inoculated samples at 1 dpi (columns AW‐AY), β‐cyclocitral‐treated P. viticola‐inoculated and β‐cyclocitral‐treated mock‐inoculated samples at 1 dpi (columns AZ‐BB), β‐cyclocitral‐treated mock‐inoculated and control mock‐inoculated samples at 1 dpi (columns BC‐BE), β‐cyclocitral‐ treated P. viticola‐inoculated and control P. viticola‐inoculated samples at 1 dpi (columns BF‐BH), control P. viticola‐inoculated and control mock‐inoculated at 6 dpi (columns BI‐BK), 2‐phenylethanol‐treated P. viticola‐inoculated and 2‐phenylethanol‐treated mock‐inoculated samples at 6 dpi (columns BL‐BN), 2‐phenylethanol‐treated mock‐inoculated and control mock‐inoculated samples at 6 dpi (columns BO‐BQ), 2‐phenylethanol‐treated P. viticola‐inoculated and control P. viticola‐inoculated samples at 6 dpi (columns BR‐BT), β‐cyclocitral‐treated P. viticola‐inoculated and β‐cyclocitral‐treated mock‐inoculated samples at 6 dpi (columns BU‐BW), β‐cyclocitral‐treated mock‐inoculated and control mock‐inoculated samples at 6 dpi (columns BX‐BZ), β‐cyclocitral‐ treated P. viticola‐inoculated and control P. viticola‐inoculated samples at 6 dpi (columns CA‐CC). Metabolic features with significant increases (UP) or decreases (DOWN) in abundance were selected with Compound Discoverer, imposing a Log2‐transformed fold change (LFC) lower than −1 or higher than 1 and a *p* value of *t*‐test lower than 0.05 in at least one pairwise comparison. Metabolic features were annotated with Compound Discoverer and the most probable chemical name (column CD), elemental formula (column CE), annotation delta mass (column CF), calculated molecular weight (column CG), fragmentation information (column CH), neutral losses (column CI), reference ion (column CJ), number of Chem Spider results (column CK), number of mzCloud results (column CL) are reported for each feature.
**Table S8.** Functional annotations of metabolic features with significant changes in abundance in leaf disks treated with 2‐phenylethanol. Grapevine leaf disks were treated with water (Control) or 2‐phenylethanol, inoculated with Plasmopara viticola (P. viticola‐inoculated) or water (Mock‐inoculated), and collected at one and six days post inoculation (dpi). Metabolic features (column D), specified by mean mass to charge ratio (m/z; column E) and mean retention time (rt) expressed as minutes (column F) were detected using ultra high pressure liquid chromatography ‐ heated electrospray ionization ‐ Orbitrap mass spectrometry (UHPLC‐HESI‐ 21 Orbitrap‐MS) analysis in negative heated electrospray ionization (HESI) mode (blue cells in column D) and positive HESI mode (red cells in column D). Metabolic features were annotated with Compound Discoverer and the most probable chemical name (column G), elemental formula (column H), annotation delta mass (column I), calculated molecular weight (column J), fragmentation information (column K), neutral losses (column L), reference ion (column M), number of Chem Spider results (column N), number of mzCloud results (column O) are reported for each feature. Entry codes of Kyoto Encyclopedia of Genes and Genomes (KEGG) database were obtained with MetaboAnalyst imposing an error acceptance of 3 ppm (column P). Metabolic features with significant increases (UP) or decreases (DOWN) in abundance were selected with Compound Discoverer, imposing a Log2‐transformed fold change (LFC) lower than −1 or higher than 1 and a *p* value of *t*‐test lower than 0.05 in at least one pairwise comparison (columns Q‐AB), such as between control P. viticola‐inoculated and control mock‐inoculated samples, 2‐phenylethanol‐treated P. viticola‐inoculated and 2‐phenylethanol‐treated mock‐inoculated samples, 2‐phenylethanol‐treated mock‐inoculated and control mock‐inoculated samples, 2‐phenylethanol‐ treated P. viticola‐inoculated and control P. viticola‐inoculated samples at 1 dpi and 6 dpi. Annotated metabolic features with significant changes in abundance were grouped (group from 1 to 8; column C) according to the Venn diagrams (column A; Figure S7), in those modulated in one or two pairwise comparisons at 1 dpi and 6 dpi (column B). Metabolic pathway analysis was carried out using the pathway analysis tool of MetaboAnalyst based on the KEGG codes of annotated metabolic features with significant increases (groups 1, 2, 3, 4) or decreases (groups 5, 6, 7, 8) in abundance. Manually curated annotation (columns AC‐AM) was carried out for annotated metabolic features with LFC lower than −3 or higher than 3 and a *p* value of *t*‐test lower than 0.05 by searching all putative chemical names of each annotated feature found by Compound Discoverer in the PubChem (https://pubchem.ncbi.nlm.nih.gov/), ChEBI (https://www.ebi.ac.uk/chebi/), and KEGG (https://www.kegg.jp/kegg/compound/) databases, in order to retrieve the exact mass (column AE), considering the ionization mode of detection (column AF), InChI code (column AG), InChIKey code (column AH), and spectral information (database reference spectra). Additional in silico reference spectra were obtained with InChI codes in the CFM‐ID 4.0 web server (https://cfmid.wishartlab.com). Database reference spectra and in silico reference spectra were visually compared with the experimental full scan MS spectra and ddMS2 fragmentation spectra of each annotated metabolic feature, in order to select the compound annotation (annotated compound; column AC), elemental formula (column AD), molecular ions (column AI), entry code of KEGG database (column AJ), and KEGG pathway (column AK). Annotated compounds were classified into nine putative chemical classes according to the manually curated annotation (columns AL‐AM) based on the classification obtained with the ClassyFire web‐based application (https://cfb.fiehnlab.ucdavis.edu/) (columns AN‐AS). Annotation level was 22 assigned according to the Metabolomics Standard Initiative (Sumner et al. 2007 doi: 10.1007/s11306‐ 007‐0082‐2), such as identified metabolites (level 1), putatively annotated compounds (level 2), isomeric (level 2/3), putatively characterized compound classes (level 3), and unknown (level 4) (column AT).
**Table S9.** Metabolic pathway analysis results of annotated metabolic features with significant changes in abundances in leaf disks treated with 2‐phenylethanol. Grapevine leaf disks were treated with water (Control) or 2‐phenylethanol, inoculated with Plasmopara viticola (P. viticola‐inoculated) or treated with water (Mock‐inoculated), and collected at one and six days post inoculation (dpi). Metabolic features were detected with ultra high pressure liquid chromatography—heated electrospray ionization—Orbitrap mass spectrometry (UHPLC‐HESI‐Orbitrap‐MS) analysis. Metabolic features with significant changes in abundances were selected with Compound Discoverer, imposing a Log2‐transformed fold change (LFC) lower than −1 or higher than 1 and a *p* value of *t*‐test lower than 0.05 in at least one pairwise comparison, such as between control P. viticola‐ inoculated and control mock‐inoculated samples, 2‐phenylethanol‐treated P. viticola‐inoculated and 2‐phenylethanol‐treated mock‐inoculated samples, 2‐phenylethanol‐treated mock‐inoculated and control mock‐inoculated samples, 2‐phenylethanol‐treated P. viticola‐inoculated and control P. viticola‐inoculated samples at 1 dpi and 6 dpi. Metabolic pathway analysis was carried out using the pathway analysis tool of MetaboAnalyst based on the Kyoto Encyclopedia of Genes and Genomes (KEGG) codes of annotated metabolic features with changes in abundance and grouped (column A) in those with increases (groups 1, 2, 3, 4) or decreases (groups 5, 6, 7, 8) in abundance according to the Venn diagrams at 1 dpi and 6 dpi (Figure S7). Pathway name (column B), number of compounds in each pathway (column C), number of compounds expected by chance (column D), number of KEGG codes matched to each pathway (column E), and the raw *p* value (column F) are calculated on the bases of the hypergeometric test. Log transformation (column G), Holm adjusted *p* value (column H), false discovery rate (column I), and the impact obtained with the pathway topology analysis (column J) are reported for each pathway. Metabolic pathways with Holm adjusted *p* value lower than 0.05 are highlighted in bold (adjusted *p* value ≤ 0.05).
**Table S10.** Functional annotations of annotated metabolic features with significant changes in abundance in leaf disks treated with β‐cyclocitral. Grapevine leaf disks were treated with water (Control) or β‐cyclocitral, inoculated with Plasmopara viticola (P. viticola‐inoculated) or water (Mock‐inoculated), and collected at one and six days post inoculation (dpi). 23 Metabolic features (column D), specified by mean mass to charge ratio (m/z; column E) and mean retention time (rt) expressed as minutes (column F) were detected using ultra high pressure liquid chromatography—heated electrospray ionization—Orbitrap mass spectrometry (UHPLC‐HESI‐ Orbitrap‐MS) analysis in negative heated electrospray ionization (HESI) mode (blue cells in column D) and positive HESI mode (red cells in column D). Metabolic features were annotated with Compound Discoverer and the most probable chemical name (column G), elemental formula (column H), annotation delta mass (column I), calculated molecular weight (column J), fragmentation information (column K), neutral losses (column L), reference ion (column M), number of Chem Spider results (column N), number of mzCloud results (column O) are reported for each feature. Entry codes of Kyoto Encyclopedia of Genes and Genomes (KEGG) database were obtained with MetaboAnalyst imposing an error acceptance of 3 ppm (column P). Metabolic features with significant increases (UP) or decreases (DOWN) in abundance were selected with Compound Discoverer, imposing a Log2‐transformed fold change (LFC) lower than −1 or higher than 1 and a *p* value of *t*‐test lower than 0.05 in at least one pairwise comparisons (columns Q‐AB), such as between control P. viticola‐inoculated and control mock‐inoculated samples, β‐cyclocitral‐treated P. viticola‐inoculated and β‐cyclocitral‐treated mock‐inoculated samples, β‐cyclocitral‐treated mock‐inoculated and control mock‐inoculated samples, β‐cyclocitral‐treated P. viticola‐ inoculated and control P. viticola‐inoculated samples at 1 dpi and 6 dpi. Annotated metabolic features with significant changes in abundance were grouped (group from 9 to 16; column C) according to the Venn diagrams (column A; Figure S8), in those modulated in one or two pairwise comparisons at 1 dpi and 6 dpi (column B). Metabolic pathway analysis was carried out using the pathway analysis tool of MetaboAnalyst based on the KEGG codes of annotated metabolic features with significant increases (groups 9, 10, 11, 12) or decreases (groups 13, 14, 15, 16) in abundance. Manually curated annotation (columns AC‐AM) was carried out for annotated metabolic features with LFC lower than −3 or higher than 3 and a *p* value of *t*‐test lower than 0.05 by searching all putative chemical names of each annotated feature found by Compound Discoverer in the PubChem (https://pubchem.ncbi.nlm.nih.gov/), ChEBI (https://www.ebi.ac.uk/chebi/), and KEGG (https://www.kegg.jp/kegg/compound/) databases, in order to retrieve the exact mass (column AE), considering the ionization mode of detection (column AF), InChI code (column AG), InChIKey code (column AH), and spectral information (database reference spectra). Additional in silico reference spectra were obtained with InChI codes in the CFM‐ID 4.0 web server (https://cfmid.wishartlab.com). Database reference spectra and in silico reference spectra were visually compared with the experimental full scan MS spectra and ddMS2 fragmentation spectra of each annotated metabolic feature, in order to select the compound annotation (annotated compound; column AC), elemental 24 formula (column AD), molecular ions (column AI), entry code of KEGG database (column AJ), and KEGG pathway (column AK). Annotated compounds were classified into nine putative chemical classes according to the manually curated annotation (columns AL‐AM) based on the classification obtained with the ClassyFire web‐ based application (https://cfb.fiehnlab.ucdavis.edu/) (columns AN‐AS). Annotation level was assigned according to the Metabolomics Standard Initiative (Sumner et al. 2007 doi: 10.1007/s11306‐007‐0082‐2) such as identified metabolites (level 1), putatively annotated compounds (level 2), isomeric (level 2/3), putatively characterized compound classes (level 3), and unknown (level 4) (column AT).
**Table S11.** Metabolic pathway analysis results of annotated metabolic features with significant changes in abundances in leaf disks treated with β‐cyclocitral. Grapevine leaf disks were treated with water (Control) or β‐cyclocitral, inoculated with Plasmopara viticola (P. viticola‐inoculated) or water (Mock‐inoculated), and collected at one and six days post inoculation (dpi). Metabolic features were detected with ultra high pressure liquid chromatography ‐ heated electrospray ionization—Orbitrap mass spectrometry (UHPLC‐HESI‐Orbitrap‐MS) analysis. Metabolic features with significant changes in abundances were selected with Compound Discoverer, imposing a Log2‐transformed fold change (LFC) lower than −1 or higher than 1 and a *p* value of t‐ test lower than 0.05 in at least one pairwise comparison, such as between control P. viticola‐ inoculated and control mock‐inoculated samples, β‐cyclocitral‐treated P. viticola‐inoculated and β‐ cyclocitral‐treated mock‐inoculated samples, β‐cyclocitral‐treated mock‐inoculated and control mock‐inoculated samples, β‐cyclocitral‐treated P. viticola‐inoculated and control P. viticola‐ inoculated samples at 1 dpi and 6 dpi. Metabolic pathway analysis was carried out using the pathway analysis tool of MetaboAnalyst based on the Kyoto Encyclopedia of Genes and Genomes (KEGG) codes of annotated metabolic features with changes in abundance and grouped (column A) in those with increases (groups 9, 10, 11, 12) or decreases (groups 13, 14, 15, 16) in abundance according to the Venn diagrams at 1 dpi and 6 dpi (Figure S8). Pathway name (column B), number of compounds in each pathway (column C), number of compounds expected by chance (column D), number of KEGG codes matched to each pathway (column E), and the raw *p* value (column F) are calculated on the bases of the hypergeometric test. Log transformation (column G), Holm adjusted *p* value (column H), false discovery rate (column I) and the impact obtained with the pathway topology analysis (column J) are reported for each pathway. Metabolic pathways with Holm adjusted *p* value lower than 0.05 are highlighted in bold (adjusted *p* value ≤ 0.05).
**Table S12.** Correlation of gene co‐expression modules of differentially expressed transcripts with the annotated compounds. (A) Co‐expression network analysis of differentially expressed transcripts (DETs) was carried out with a weighted gene co‐expression network analysis (WGCNA; v1.72.1) using the Log2 (RPM+1) values with a soft threshold power of 24 and a minimum number of transcripts per module of 15 (Figure S10). WGCNA was used to calculate the correlation of each co‐expression module, indicated with the respective color (column A) and number (column B), with the 198 annotated compounds (column C), according to a Pearson’s correlation method (columns D–F). For each annotated compound, the chemical name (column G), KEGG code (column H), manually curated annotation (column I), elemental formula (column J), and chemical class (column K) are reported. (B) List of 198 annotated compounds with significant changes in abundance (LFC lower than −3 or higher than 3 and a *p* value of *t*‐test lower than 0.05) and classified into putative chemical classes (benzenoids, carbohydrates and conjugates, carbonyl compounds, carboxylic acids and derivatives, indoles and derivatives, lipids and lipid‐like compounds, phenylpropanoids, terpenoids, and unknown) by the manually curated annotation.

## Data Availability

Raw RNA reads were deposited at the Sequence Read Archive of the NCBI (https://www.ncbi.nlm. nih.gov/sra) under the BioProject number PRJNA871393. Metabolomic data are reported as Supporting Information.
