# Supplementary material for: Volatile 2‐Phenylethanol and β‐Cyclocitral Trigger Defense‐Related Transcriptional and Metabolic Changes in Grapevine Leaves Against Downy Mildew
Source: Physiol Plant. 2025 Jul 22;177(4):e70412. doi: 10.1111/ppl.70412 (PMC12284132; doi:10.1111/ppl.70412)
Supplement: Supplementary file 1 — Figure S1. Untargeted metabolomics workflow of Compound Discoverer. Grapevine leaf disk samples were analyzed using ultra‐high pressure liquid chromatography—heated electrospray ionization—Orbitrap mass spectrometry analysis. Full scan MS data and ddMS2 fragmentation data were processed using an untargeted metabolomics workflow (Untargeted Metabolomics with Statistics Detect Unknown with ID using Online Database and mzLogic) on Compound Discoverer. The nodular workflow involved spectrum selection (Select Spectra), chromatographic alignment (Align Retention Times), metabolic feature detection (Detect Compounds, Group Compounds, Fill Gaps), peak area normalization according to quality control samples (Apply SERRF QC Correction, Mark Background Compounds, Normalize Areas), chemical annotation of metabolic features (Assign Compound Annotations, Predict Compositions, Search ChemSpider, Search mzCloud nodes, Search Mass Lists, Apply mzLogic node and Search Neutral Losses). Metabolic features with significant changes in abundances were selected, imposing a Log2‐transformed fold change lower than −1 or higher than 1 and a p value of t‐test lower than 0.05 in seven pairwise comparisons for each time point (Differential Analysis). Figure S2. Multi‐dimensional scaling (MDS) plot of transcriptomic data. Grapevine leaf disks were treated with water (Control; blue), 2‐phenylethanol (2PE‐treated; red), or β‐cyclocitral (βCC‐treated; green), inoculated with Plasmopara viticola (P. viticola‐inoculated; solid symbols) or water (Mock‐ inoculated). Three replicates (pool of ten leaf disks each) for each treatment were collected at one and six days post inoculation (dpi) and subjected to RNA‐Seq analysis. First and second dimension (A) or first and third dimension (B) of MDS plot were obtained with the EdgeR v3.38.4 tool on normalized counts of 20,667 active transcripts detected in grapevine samples 4. Figure S3. RNA‐Seq data validation by qPCR. Heatmaps of Log2‐transformed fold change [file PPL-177-e70412-s002.pdf]

Volatile 2-phenylethanol and β-cyclocitral trigger defense-related transcriptional and metabolic changes in grapevine leaves against downy mildew

Sara Avesani[1,2,3], Valentina Lazazzara[3], Matteo Buti[4], Michael Oberhuber[2], Peter Robatscher[2], Michele Perazzolli[1,3,*]

[1] Center for Agriculture Food Environment (C3A), University of Trento, Via E. Mach 1, 38098 San Michele all'Adige, Italy

[2] Laboratory for Flavours and Metabolites, Laimburg Research Centre, Laimburg 6, Pfatten (Vadena), 39040 Auer (Ora), Italy

[3] Research and Innovation Centre, Fondazione Edmund Mach, Via E. Mach 1, 38098 San Michele all'Adige, Italy

[4] Department of Agriculture, Food, Environmental and Forestry Sciences (DAGRI), University of Florence, 50144 Florence, Italy

[*] Correspondence

Michele Perazzolli

E-mail: michele.perazzolli@unitn.it

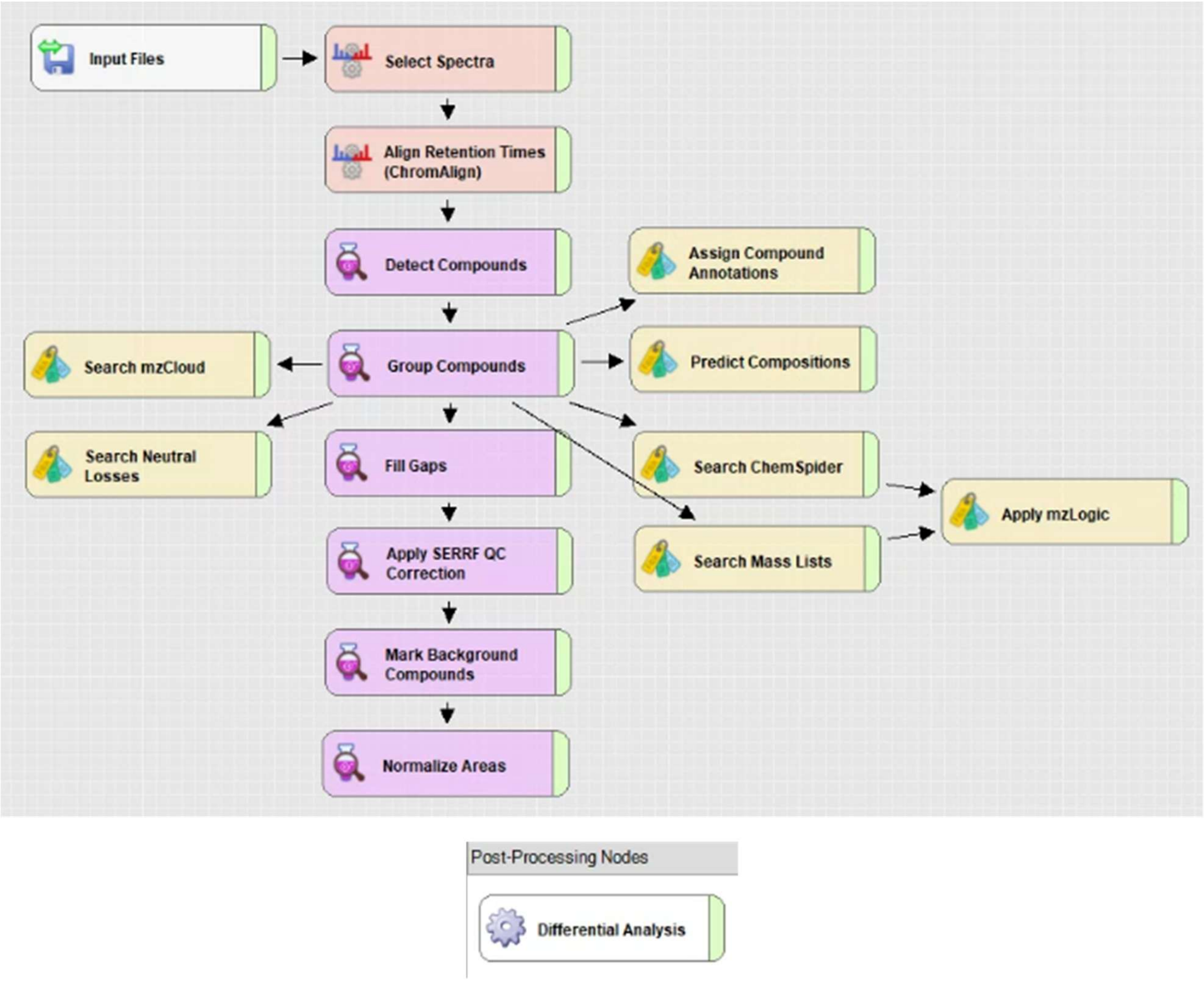

Figure S1. Untargeted metabolomics workflow of Compound Discoverer. Grapevine leaf disk samples were analyzed using ultra-high pressure liquid chromatography - heated electrospray ionization - Orbitrap mass spectrometry analysis. Full scan MS data and ddMS2 fragmentation data were processed using an untargeted metabolomics workflow (Untargeted Metabolomics with Statistics Detect Unknown with ID using Online Database and mzLogic) on Compound Discoverer. The nodular workflow involved spectrum selection (Select Spectra), chromatographic alignment (Align Retention Times), metabolic feature detection (Detect Compounds, Group Compounds, Fill Gaps), peak area normalization according to quality control samples (Apply SERRF QC Correction, Mark Background Compounds, Normalize Areas), chemical annotation of metabolic features (Assign Compound Annotations, Predict Compositions, Search ChemSpider, Search mzCloud nodes, Search Mass Lists, Apply mzLogic node and Search Neutral Losses). Metabolic features with significant changes in abundances were selected, imposing a Log_2-transformed fold change lower than -1 or higher than 1 and a *P*-value of *t*-test lower than 0.05 in seven pairwise comparisons for each time point (Differential Analysis).

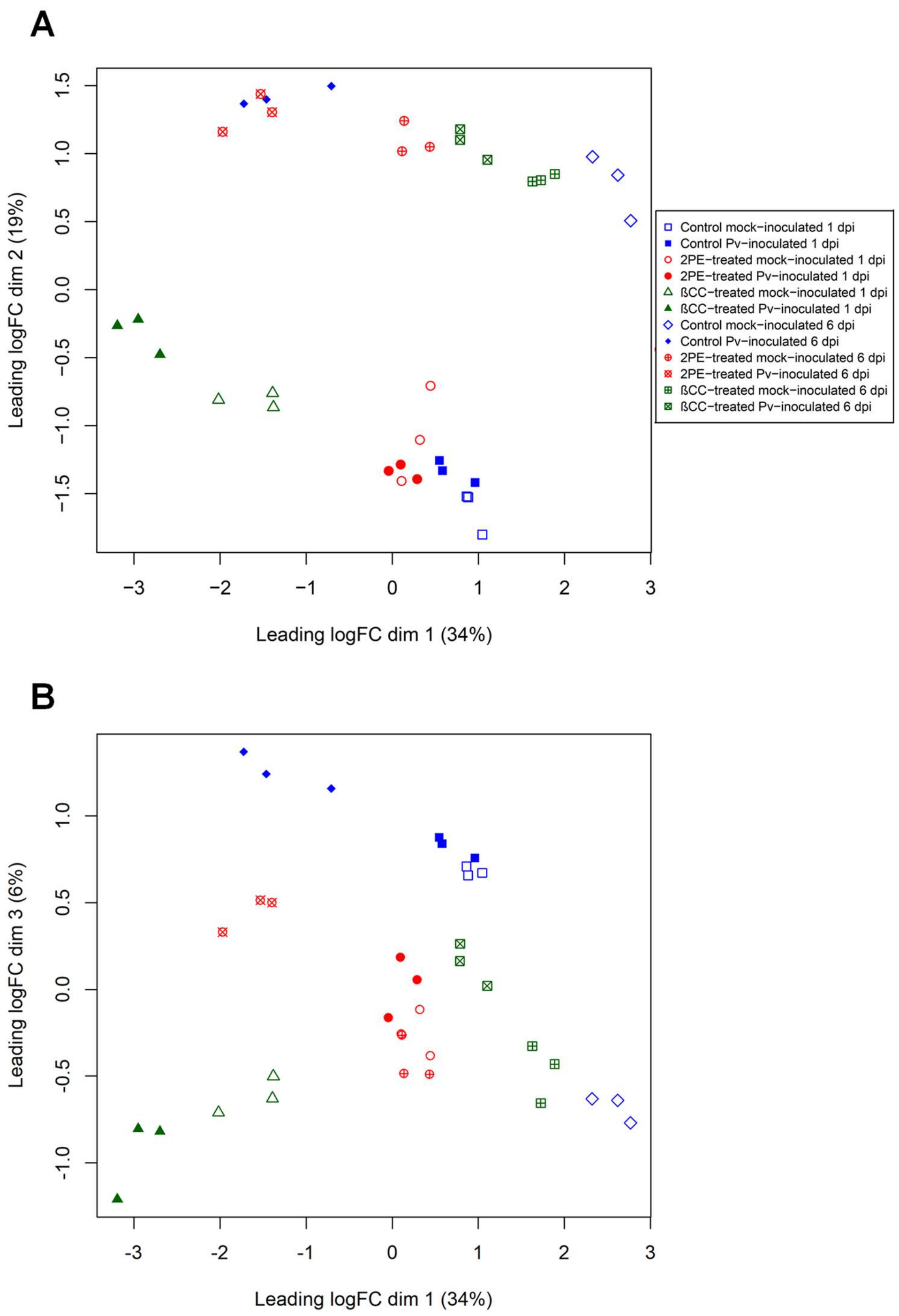

Figure S2. Multi-dimensional scaling (MDS) plot of transcriptomic data. Grapevine leaf disks were treated with water (Control; blue), 2-phenylethanol (2PE-treated; red), or β-cyclocitral (βCC-treated; green), inoculated with *Plasmopara viticola* (*P. viticola*-inoculated; solid symbols) or water (Mock-inoculated). Three replicates (pool of ten leaf disks each) for each treatment were collected at one and six days post inoculation (dpi) and subjected to RNA-Seq analysis. First and second dimension (A) or first and third dimension (B) of MDS plot were obtained with the EdgeR v3.38.4 tool on normalized counts of 20,667 active transcripts detected in grapevine samples.

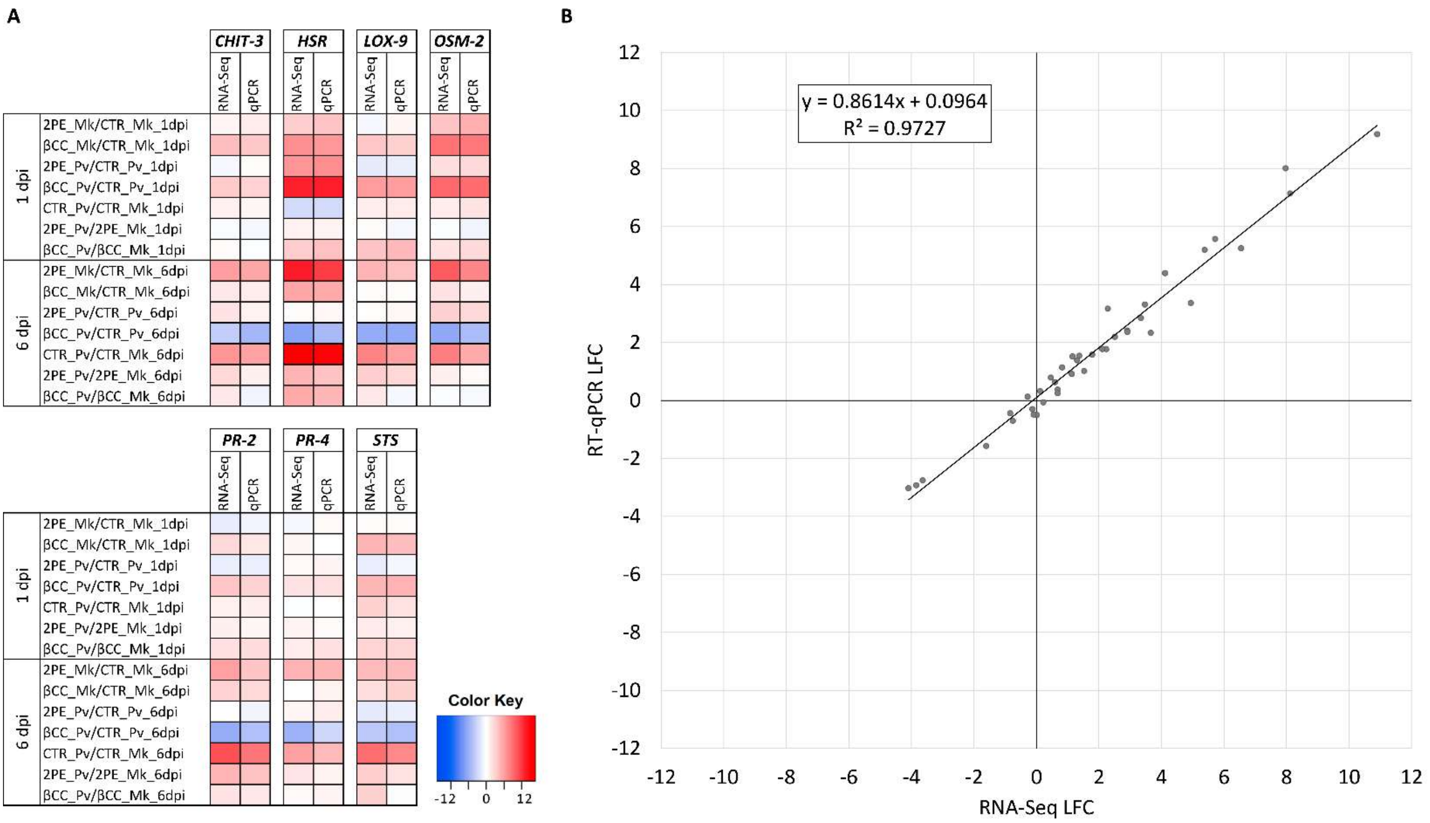

Figure S3. RNA-Seq data validation by qPCR. Heatmaps of Log$_2$-transformed fold change (LFC) values assessed by RNA-Seq and qPCR are reported according to the color scale legend for transcript encoding chitinase 3 (*CHIT-3*), hypersensitive response-related gene (*HSR*), lipoxygenase 9 (*LOX-9*), osmotin 2 (*OSM-2*), pathogenesis-related (PR) protein 2 (*PR-2*) and 4 (*PR-4*), and stilbene synthase gene (*STS*) in the pairwise comparisons of grapevine leaf disks treated with water (Control; CTR), 2-phenylethanol (2PE), or β-cyclocitral (βCC), inoculated with *Plasmopara viticola* (Pv) or water (Mock-inoculated; Mk) and collected at one or six days post inoculation (dpi) (A). A scatter plot of LFC values of RNA-Seq and qPCR analyses is reported with the regression line equation and the R^2 coefficient value (B).

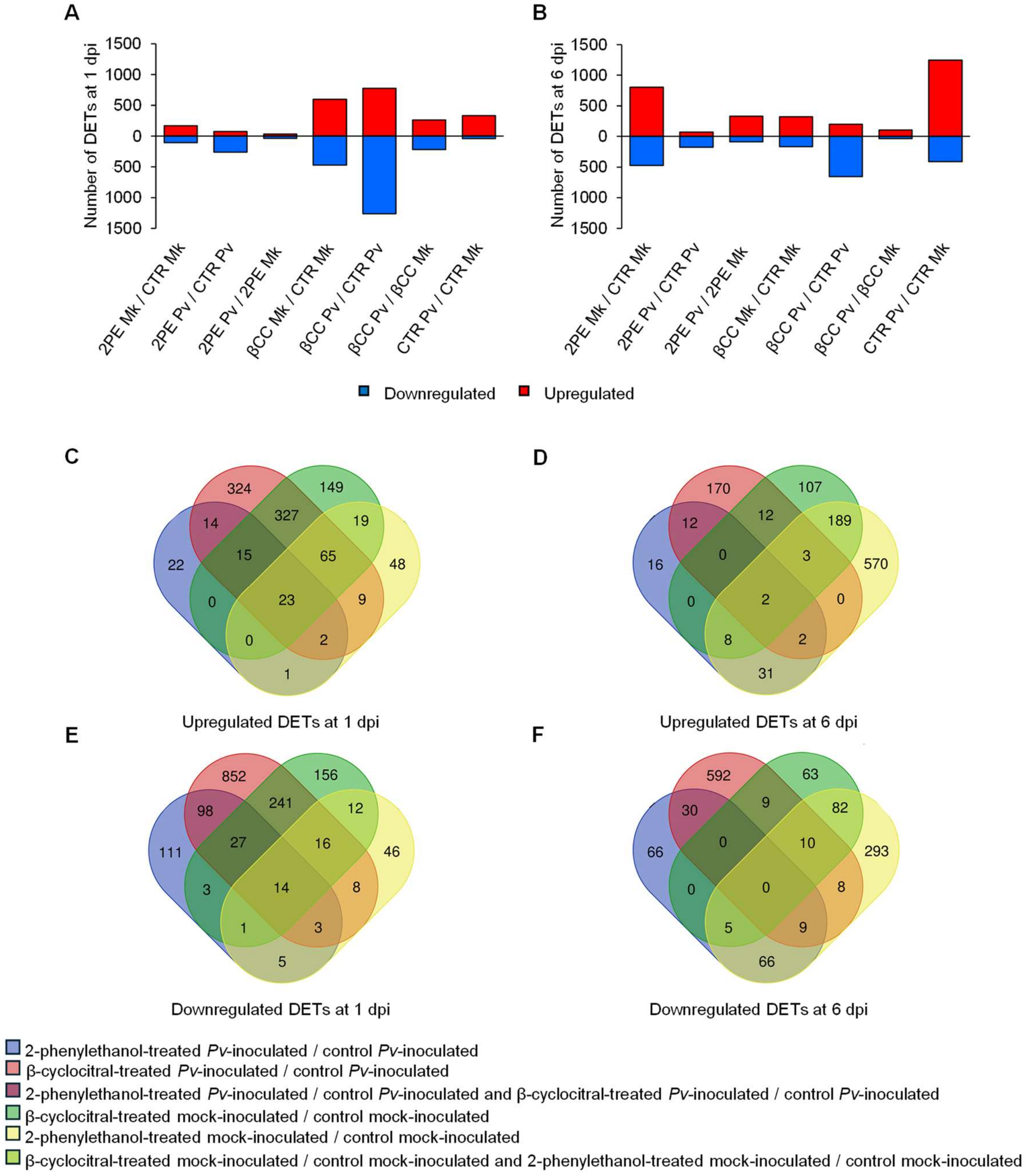

Figure S4. Differential expression analysis results. Numbers of upregulated (red bars) and downregulated (blue bars) differentially expressed transcripts (DETs; $\log_2$-transformed fold change lower than -2 or higher than 2, and false discovery rate lower than 0.05) are reported for each pairwise comparison of grapevine leaf disks treated with water (Control; CTR), 2-phenylethanol (2PE), or β-cyclocitral (βCC), inoculated with *Plasmopara viticola* (*P. viticola*-inoculated; Pv) or water (Mock-inoculated; Mk), and collected at one (A) and six (B) days post inoculation (dpi). Venn diagrams summarize the distribution of upregulated (C, D) and downregulated (E, F) DETs at 1 dpi (C, E) and 6 dpi (D, F) in the pairwise comparisons specified by the color legend.

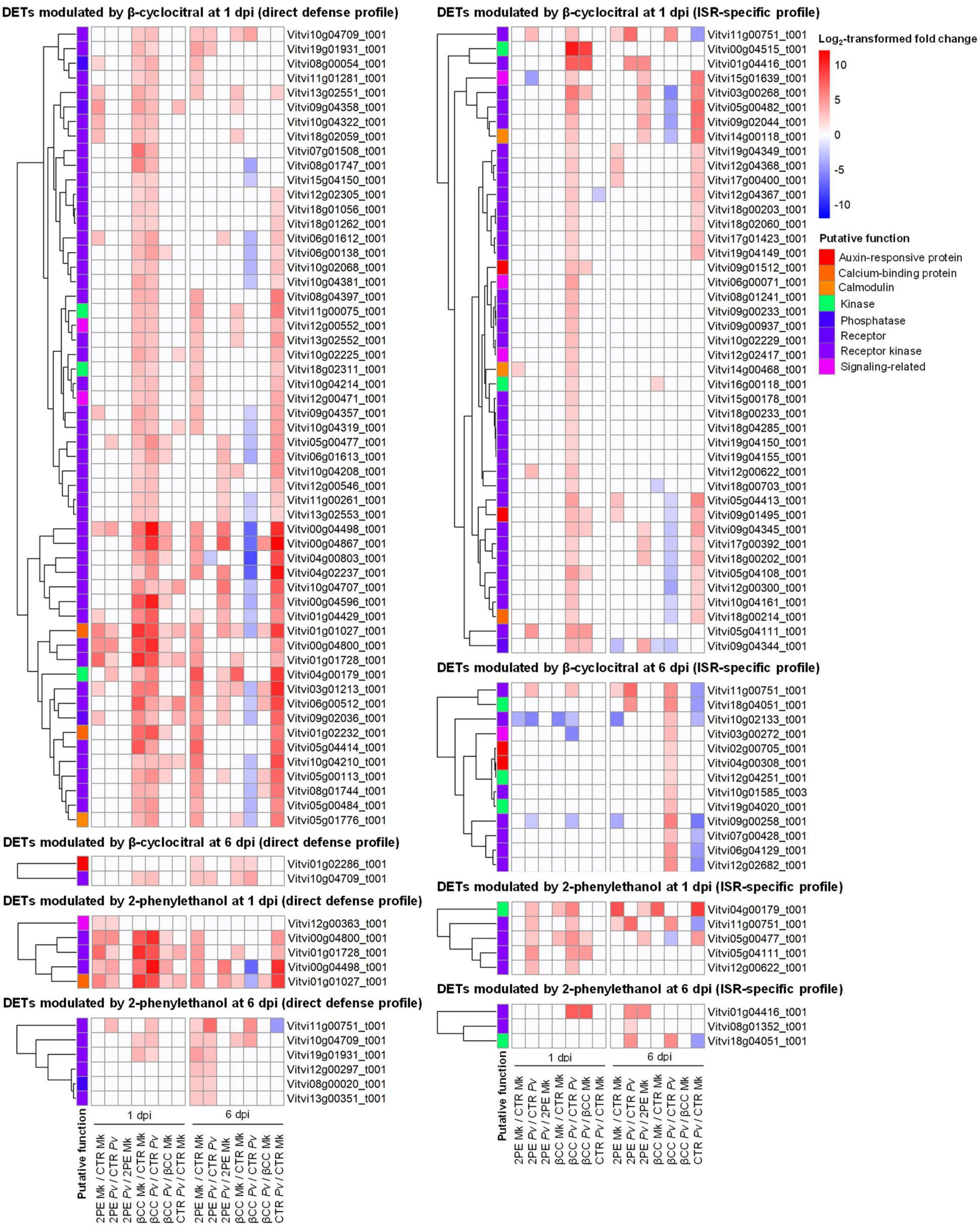

Figure S5. Heatmaps of differentially expressed transcripts (DETs) involved in signal transduction. Log$_2$-transformed fold change values (color legend; LFC) of DETs (LFC lower than -2 or higher than 2 and false discovery rate lower than 0.05) are reported for pairwise comparisons of grapevine leaf disks treated with water (Control; CTR), 2-phenylethanol (2PE), or β-cyclocitral (βCC), inoculated with *Plasmopara viticola* (Pv) or water (Mk; mock-inoculated) and collected at one or six days post inoculation (dpi). Putative functions (color legend) of DETs with direct defense profile (modulation

in VOC-treated compared to control leaf disks in mock-inoculated and *P. viticola*-inoculated samples) or ISR-specific profile (modulation in VOC-treated compared to control leaf disks only in *P. viticola*-inoculated samples) were assigned according to the manually curated annotation based on the protein description search and GO biological process annotations.

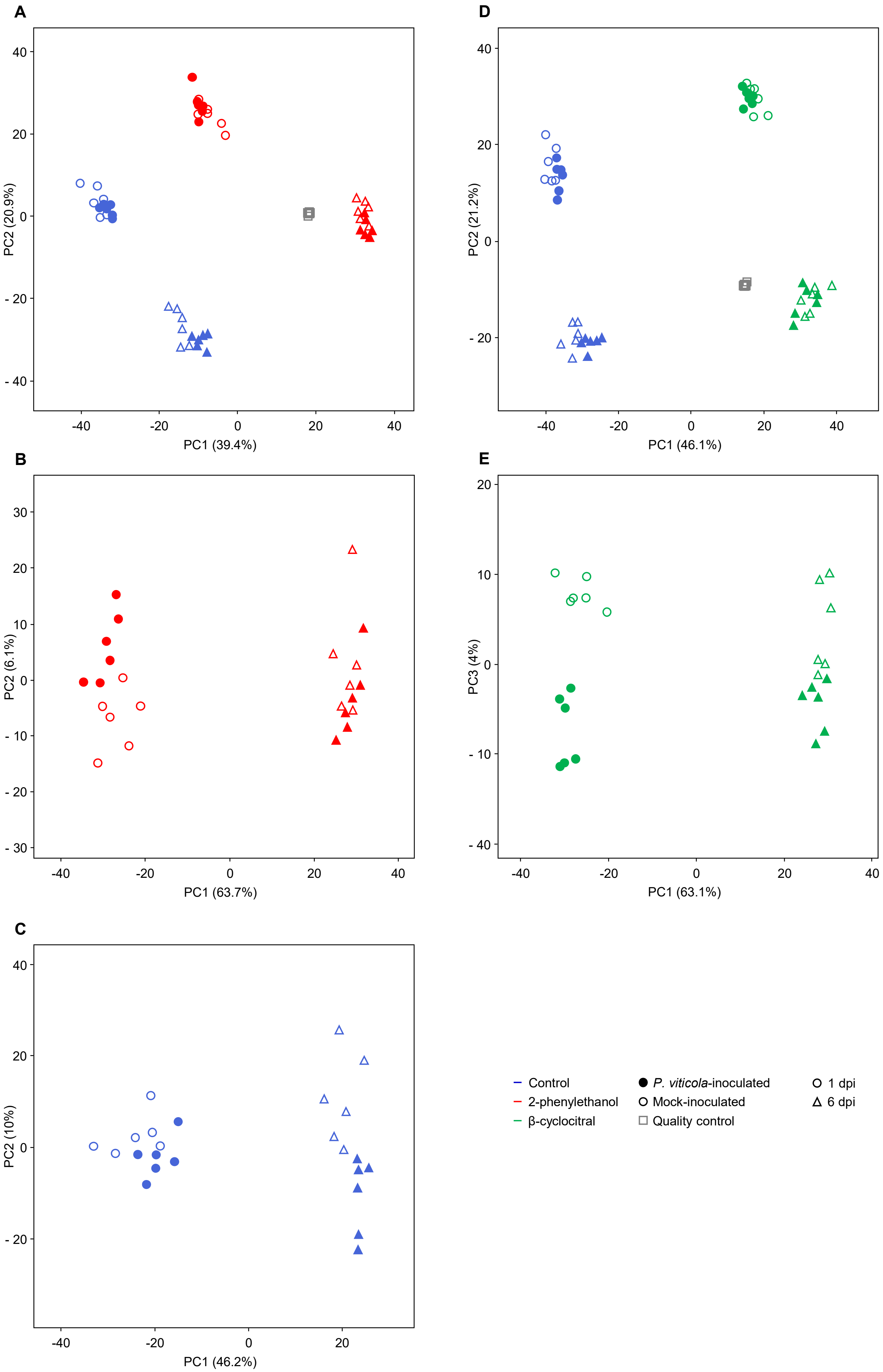
A
B
C
D
E
PC1 (39.4%)
PC2 (20.9%)
PC1 (63.7%)
PC2 (6.1%)
PC1 (46.2%)
PC2 (10%)
PC1 (46.1%)
PC2 (21.2%)
PC1 (63.1%)
PC3 (4%)
Control
2-phenylethanol
β-cyclocitral
P. viticola-inoculated
Mock-inoculated
Quality control
1 dpi
6 dpi

Figure S6. Principal component analysis (PCA) of metabolomic data. Grapevine leaf disks were treated with water (Control; blue), 2-phenylethanol (red), or β-cyclocitral (green), inoculated with *Plasmopara viticola* (*P. viticola*-inoculated; solid symbols) or water (Mock-inoculated; open symbols). Six replicates (pool of ten leaf disks each) for each treatment were collected at one (circles) and six (triangles) days post inoculation (dpi) and subjected to ultra high pressure liquid chromatography - heated electrospray ionization - Orbitrap mass spectrometry (UHPLC-HESI-Orbitrap-MS) analysis. PCA was obtained with MetaboAnalyst on 13,475 metabolic features detected in grapevine samples. Quality control samples (gray squares) were obtained from the homogenization of equal aliquots of each sample.

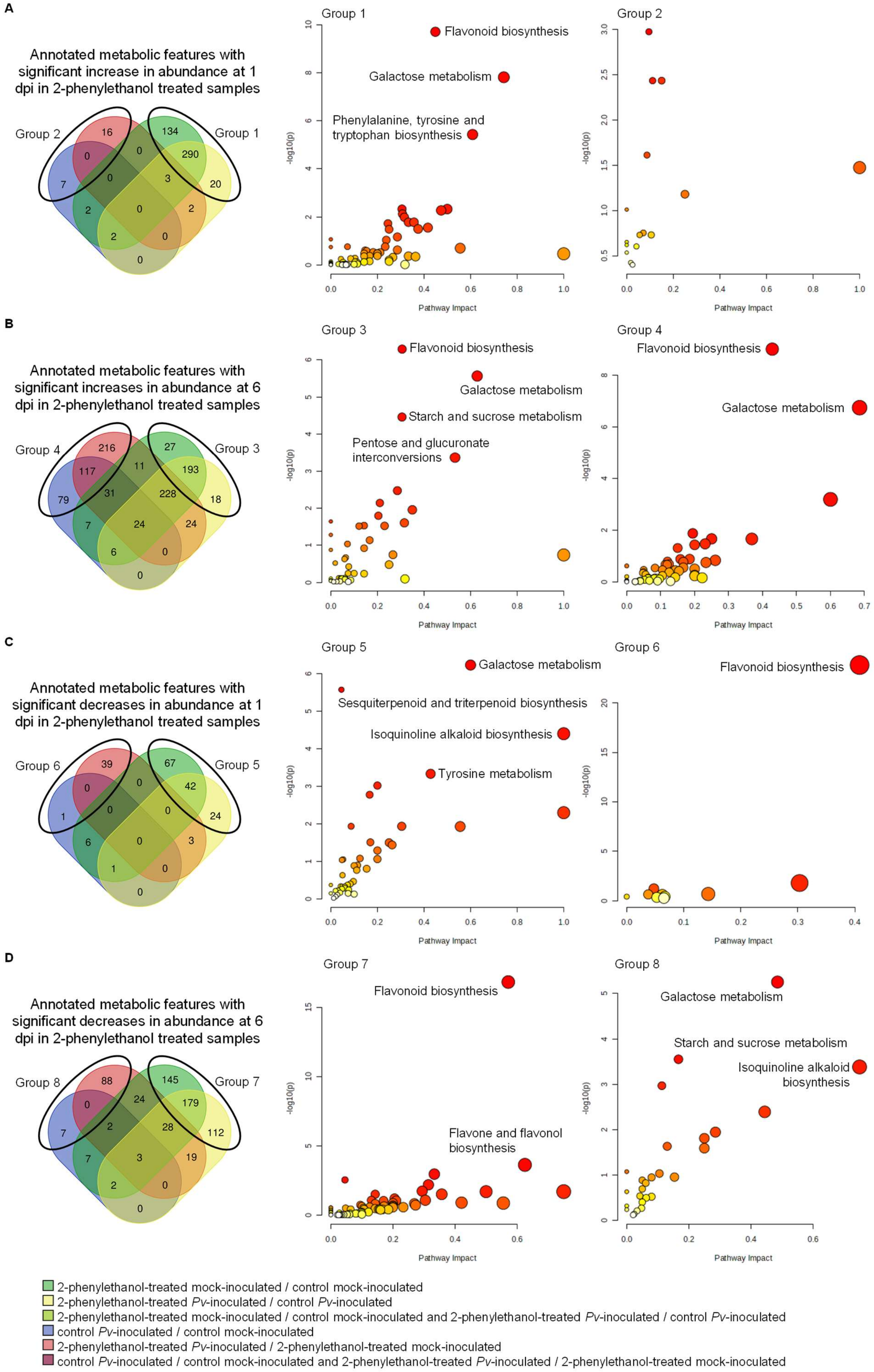
A
Annotated metabolic features with significant increase in abundance at 1 dpi in 2-phenylethanol treated samples
Group 2
Group 1
Group 1
Flavonoid biosynthesis
Galactose metabolism
Phenylalanine, tyrosine and tryptophan biosynthesis
Group 2
-log10(p)
Pathway Impact
B
Annotated metabolic features with significant increases in abundance at 6 dpi in 2-phenylethanol treated samples
Group 4
Group 3
Group 3
Flavonoid biosynthesis
Galactose metabolism
Starch and sucrose metabolism
Pentose and glucuronate interconversions
Group 4
Flavonoid biosynthesis
Galactose metabolism
C
Annotated metabolic features with significant decreases in abundance at 1 dpi in 2-phenylethanol treated samples
Group 6
Group 5
Group 5
Galactose metabolism
Sesquiterpenoid and triterpenoid biosynthesis
Isoquinoline alkaloid biosynthesis
Tyrosine metabolism
Group 6
Flavonoid biosynthesis
D
Annotated metabolic features with significant decreases in abundance at 6 dpi in 2-phenylethanol treated samples
Group 8
Group 7
Group 7
Flavonoid biosynthesis
Flavone and flavonol biosynthesis
Group 8
Galactose metabolism
Starch and sucrose metabolism
Isoquinoline alkaloid biosynthesis
2-phenylethanol-treated mock-inoculated / control mock-inoculated
2-phenylethanol-treated Pv-inoculated / control Pv-inoculated
2-phenylethanol-treated mock-inoculated / control mock-inoculated and 2-phenylethanol-treated Pv-inoculated / control Pv-inoculated
control Pv-inoculated / control mock-inoculated
2-phenylethanol-treated Pv-inoculated / 2-phenylethanol-treated mock-inoculated
control Pv-inoculated / control mock-inoculated and 2-phenylethanol-treated Pv-inoculated / 2-phenylethanol-treated mock-inoculated

Figure S7. Metabolic pathway analysis of annotated metabolic features with significant changes in abundances in leaf disks treated with 2-phenylethanol. Venn diagrams summarize the distribution of annotated metabolic features with significant increases (A, B) and decreases (C, D) in abundance (Log_2-transformed fold change lower than -1 or higher than 1, and a *P*-value of *t*-test lower than 0.05) in the pairwise comparisons (color legend) of grapevine leaf disks treated with water (Control) or 2-phenylethanol, inoculated with *Plasmopara viticola* (*Pv*-inoculated) or water (Mock-inoculated), and collected at one (A, C) and six (B, D) days post inoculation (dpi). Metabolic pathway analysis was carried out using the pathway analysis tool of MetaboAnalyst based on the Kyoto Encyclopedia of Genes and Genomes (KEGG) data of annotated metabolic features with increases (groups 1, 2, 3, and 4) or decreases (groups 5, 6, 7, and 8) in abundance reported in the Venn diagrams and affected mainly by 2-phenylethanol treatment (groups 1, 3, 5, and 7) or *P. viticola* inoculation (groups 2, 4, 6, and 8). Pathways are arranged according to *P*-values (y-axis) of pathway enrichment analysis and pathway impact values (x-axis) of pathway topology analysis. The node color and size of each pathway are determined by the *P*-value (red color with the lowest *P*-value) and impact value (diameter proportional to the impact), respectively. Enriched metabolic pathways (adjusted *P*-value ≤ 0.05) are reported for each chart.

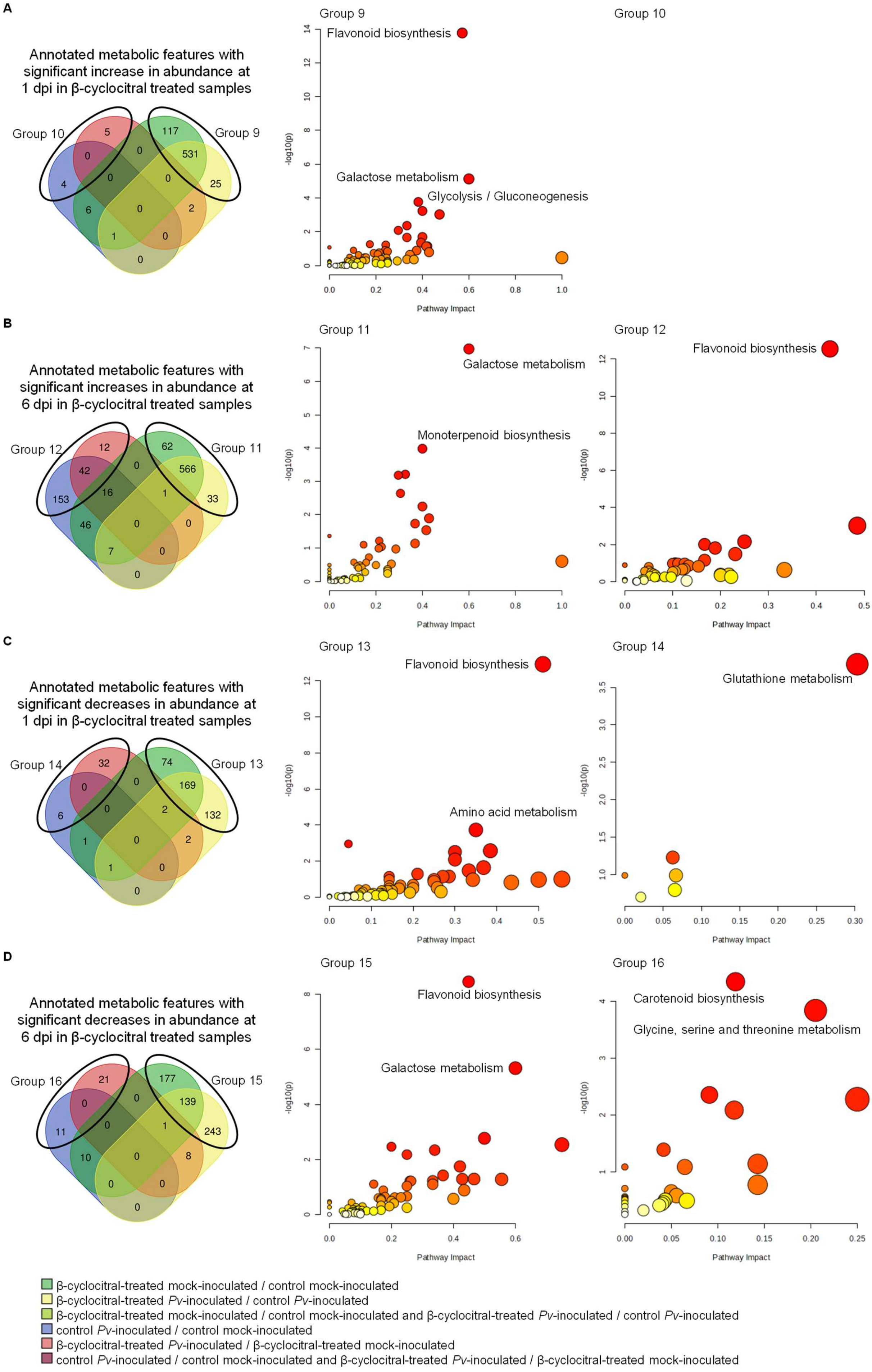
A
Annotated metabolic features with significant increase in abundance at 1 dpi in β-cyclocitral treated samples
Group 10
Group 9
Flavonoid biosynthesis
Galactose metabolism
Glycolysis / Gluconeogenesis
-log10(p)
Pathway Impact
B
Annotated metabolic features with significant increases in abundance at 6 dpi in β-cyclocitral treated samples
Group 12
Group 11
Galactose metabolism
Monoterpenoid biosynthesis
Flavonoid biosynthesis
C
Annotated metabolic features with significant decreases in abundance at 1 dpi in β-cyclocitral treated samples
Group 14
Group 13
Flavonoid biosynthesis
Amino acid metabolism
Glutathione metabolism
D
Annotated metabolic features with significant decreases in abundance at 6 dpi in β-cyclocitral treated samples
Group 16
Group 15
Flavonoid biosynthesis
Galactose metabolism
Carotenoid biosynthesis
Glycine, serine and threonine metabolism
β-cyclocitral-treated mock-inoculated / control mock-inoculated
β-cyclocitral-treated Pv-inoculated / control Pv-inoculated
β-cyclocitral-treated mock-inoculated / control mock-inoculated and β-cyclocitral-treated Pv-inoculated / control Pv-inoculated
control Pv-inoculated / control mock-inoculated
β-cyclocitral-treated Pv-inoculated / β-cyclocitral-treated mock-inoculated
control Pv-inoculated / control mock-inoculated and β-cyclocitral-treated Pv-inoculated / β-cyclocitral-treated mock-inoculated

Figure S8. Metabolic pathway analysis of annotated metabolic features with significant changes in abundances in leaf disks treated with β-cyclocitral. Venn diagrams summarize the distribution of annotated metabolic features with significant increases (A, B) and decreases (C, D) in abundance (Log_2-transformed fold change lower than -1 or higher than 1, and a *P*-value of *t*-test lower than 0.05) in the pairwise comparisons (color legend) of grapevine leaf disks treated with water (Control) or β-cyclocitral, inoculated with *Plasmopara viticola* (*Pv*-inoculated) or water (Mock-inoculated), and collected at one (A, C) and six (B, D) days post inoculation (dpi). Metabolic pathway analysis was carried out using the pathway analysis tool of MetaboAnalyst based on the Kyoto Encyclopedia of Genes and Genomes (KEGG) data of annotated metabolic features with increases (groups 9, 10, 11, and 12) or decreases (groups 13, 14, 15, and 16) in abundance reported in the Venn diagrams and affected mainly by β-cyclocitral treatment (groups 9, 11, 13, 15) or *P. viticola* inoculation (groups 10, 12, 14, 16). Pathways are arranged according to *P*-values (y-axis) of pathway enrichment analysis and pathway impact values (x-axis) of pathway topology analysis. The node color and size of each pathway are determined by the *P*-value (red color with the lowest *P*-value) and impact value (diameter proportional to the impact), respectively. Enriched metabolic pathways (adjusted *P*-value ≤ 0.05) are reported for each chart.

A **β-cyclocitral**

Authentic reference standard measured rt: 11.27 min
Grapevine leaf disk sample measured rt: 11.273 min

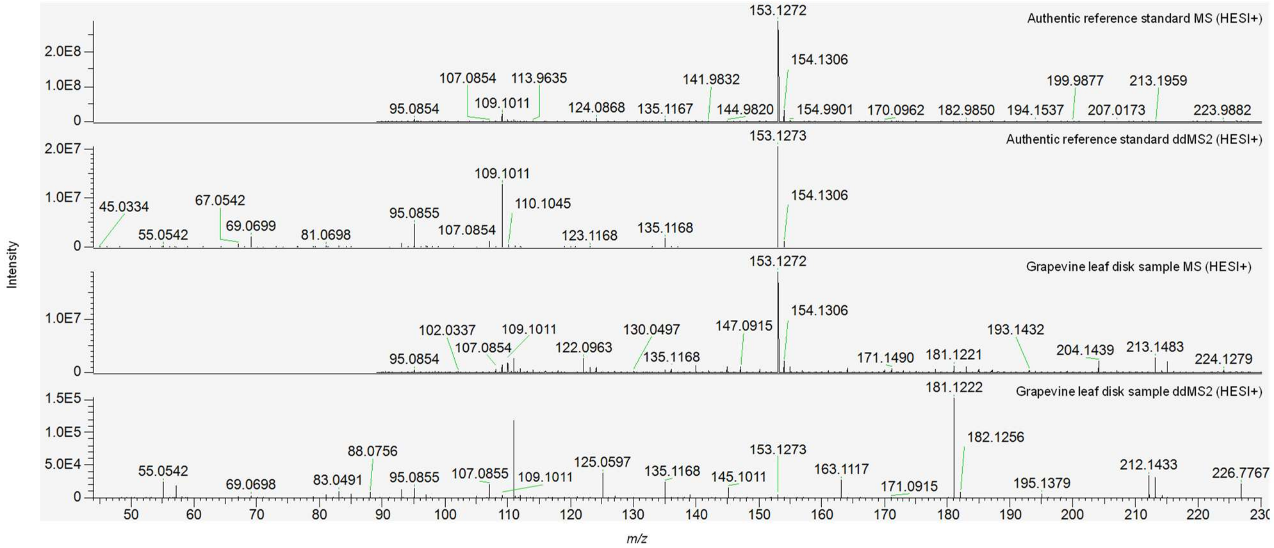

B **β-cyclocitric acid**

Authentic reference standard measured rt: 9.08 min
Grapevine leaf disk sample measured rt: 9.076 min

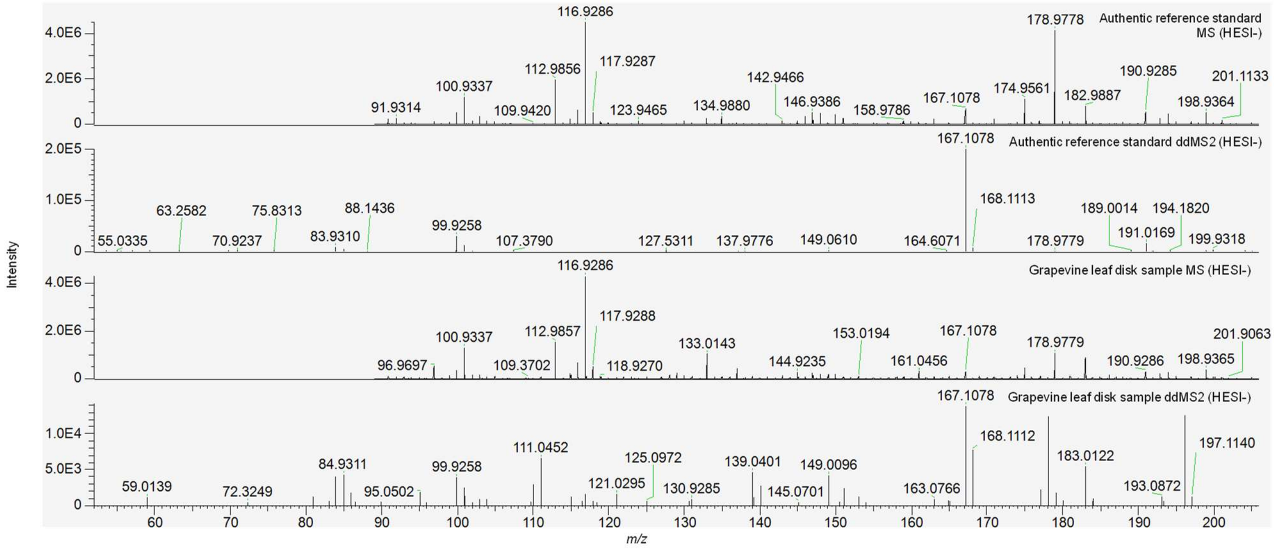

C **Geranyl pyrophosphate**

Authentic reference standard measured rt: 6.65 min
Grapevine leaf disk sample measured rt: 6.665 min

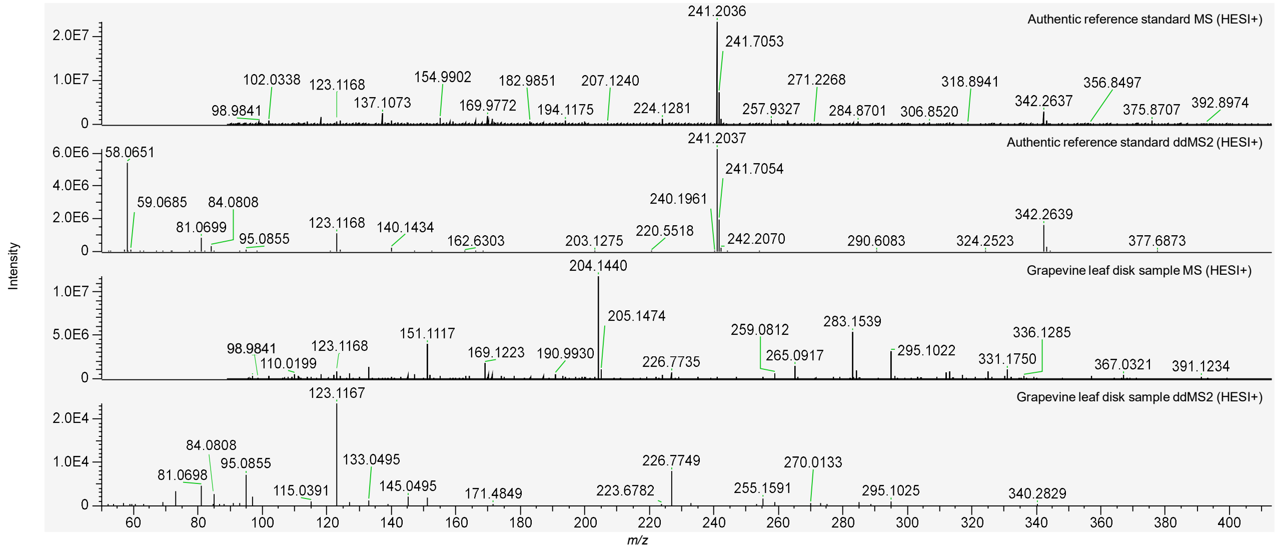

D ***trans*-resveratrol**

Authentic reference standard measured rt: 7.04 min
Grapevine leaf disk sample measured rt: 7.051 min

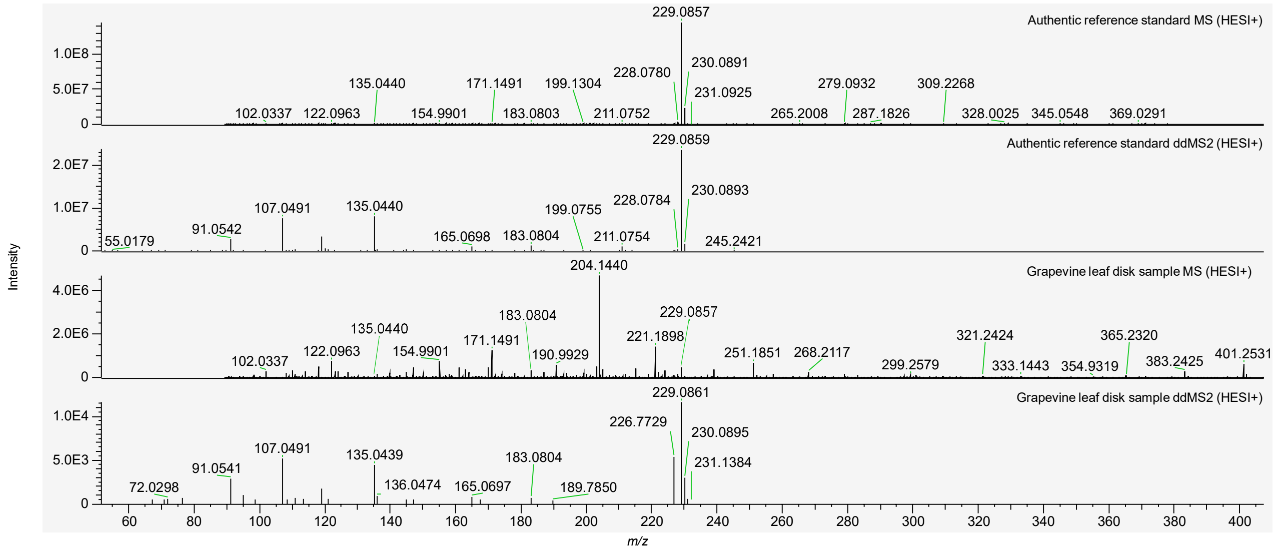

Figure S9. Comparison of the experimental mass spectra of annotated compounds found in grapevine leaf samples with the experimental mass spectra of the corresponding authentic reference standards: β-cyclocitral (A), β-cyclocitric acid (B), geranyl pyrophosphate (C), and *trans*-resveratrol (D). The comparison of full scan mass (MS) spectra and data-dependent tandem mass spectrometry (ddMS2) fragmentation spectra, retention time, and heated electrospray ionization (HESI) mode is reported for each compound.

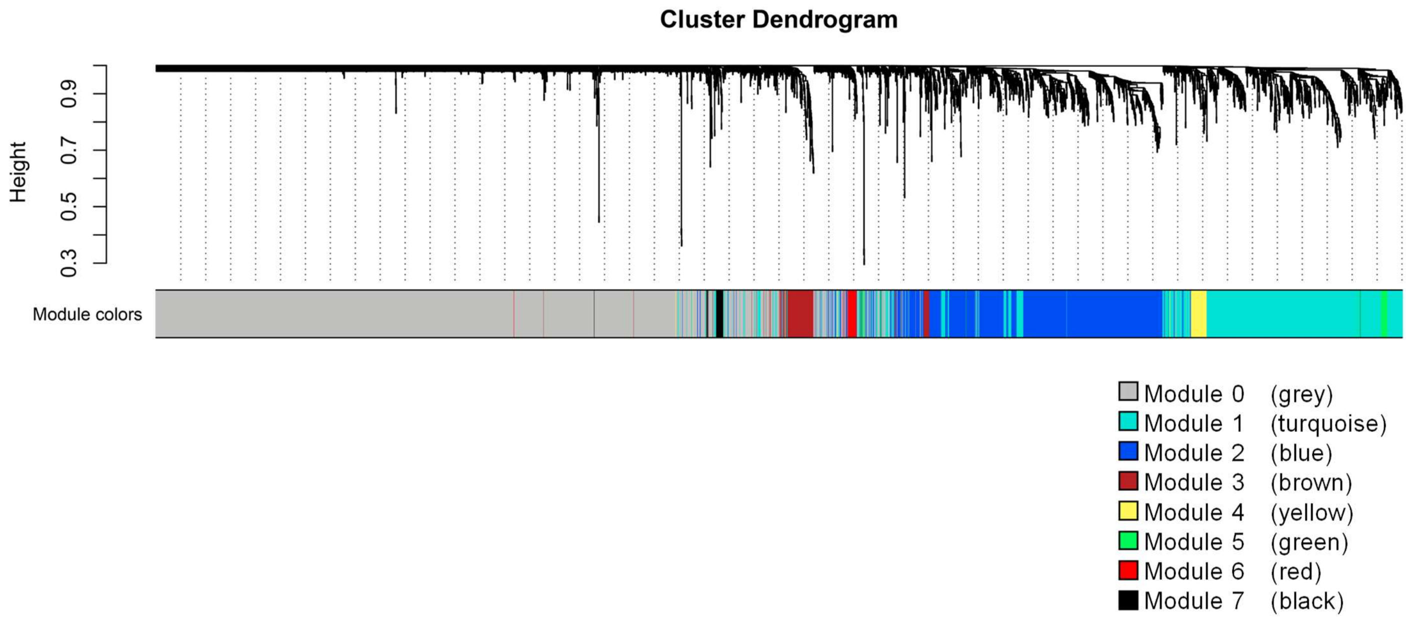

Figure S10. Co-expression network analysis of differentially expressed transcripts (DETs). Weighted co-expression network analysis (WGCNA) of DETs was carried out using the Log_2 (RPM+1), and the height of the cluster dendrogram represents the dissimilarity between transcripts within the seven co-expression modules. The correlation of co-expression modules with the annotated compounds revealed relationships (Pearson's coefficient ≥ 0.6 and p-value ≤ 0.05) between the modules of DETs and the profiles of annotated compounds (Table S12).

Legends of Tables

Table S1. Primer sequences of grapevine genes analyzed by quantitative real-time PCR.
Gene name (column A), gene abbreviation (column B), accession numbers in the National Center for Biotechnology Information (NCBI) database (http://www.ncbi.nlm.nih.gov; column C), and transcript ID (column D) of the grapevine genome (PN40024.v4.1; https://integrape.eu/resources/genes-genomes/genome-accessions) are reported for each gene. Sequences of the forward primer (column E) and reverse primer (column F) are indicated for each gene with the respective reference (column G).

Table S2. RNA-Seq data elaboration statistics.
RNA-Seq data of grapevine leaf disk samples treated with water (Control), 2-phenylethanol (2PE), or β-cyclocitral (βCC), inoculated with *Plasmopara viticola* (Pv-inoculated) or water (mock-inoculated), and collected in triplicate (named rep 1, rep 2, and rep 3) at one and six days post inoculation (dpi). The number of paired-end raw reads (column E), the number and the percentage of filtered paired-end reads after quality filtering (filtered read pairs; columns F and G), and the number and the percentage of paired-end reads aligned to the grapevine reference genome (*V. vinifera* PN40024.v4; mapped read pairs; columns H and I) are reported for each sample.

Table S3. Expression levels of grapevine transcripts.
For each grapevine transcript (*V. vinifera* PN40024.v4), expression levels are reported as fragments per kilobase million (RPM) for each sample of grapevine leaf disks treated with water (Control; CTR), 2-phenylethanol (2PE), or β-cyclocitral (βCC), inoculated with *Plasmopara viticola* (*P. viticola*-inoculated; Pv) or water (Mock-inoculated; Mk), and collected in triplicate (named rep 1, rep 2, and rep 3) at one and six days post inoculation (dpi; columns B-AK).

Table S4. Differential expression analysis results.
Grapevine leaf disks were treated with water (Control; CTR), 2-phenylethanol (2PE), or β-cyclocitral (βCC), inoculated with *Plasmopara viticola* (*P. viticola*-inoculated; Pv) or water (Mock-inoculated; Mk), and collected in triplicate at one and six days post inoculation (dpi). Differential expression analysis was carried out on active transcripts (RPM >1 in at least two libraries) with the likelihood ratio test for seven pairwise comparisons for each timepoint (columns A, B, and C) imposing a Log_2-transformed fold change (LFC) lower than -2 or higher than 2 and a false discovery rate (FDR) lower than 0.05. The number of downregulated transcripts (Column D), upregulated transcript (Column E), and total modulated transcripts (Column F) are reported for each pairwise comparison.

Table S5. Expression levels and functional annotations of differentially expressed transcripts (DETs). Grapevine leaf disks were treated with water (Control; CTR), 2-phenylethanol (2PE), or β-cyclocitral (βCC), inoculated with *Plasmopara viticola* (*P. viticola*-inoculated; Pv) or water (Mock-inoculated; Mk), and collected at one and six days post inoculation (dpi). Differentially expressed transcripts (DETs) were obtained imposing a Log_2-transformed fold change (LFC) lower than -2 or higher than 2 and a false discovery rate (FDR) lower than 0.05 with the likelihood ratio test for seven pairwise comparisons for each timepoint.

For each DET (*V. vinifera* PN40024.v4), LFC values and FDR values are reported for each pairwise comparison (columns B-AC). DETs were grouped in transcript with direct defense profile (columns AD, AF, AH, AJ, AL, AN, AP, AR; upregulation or downregulation in the pairwise comparisons between 2-phenylethanol- or β-cyclocitral-treated compared to control leaf disks in both mock-inoculated and *P. viticola*-inoculated samples at 1 dpi or 6 dpi) or ISR-specific profile (columns AE, AG, AI, AK, AM, AO, AQ, AS; upregulation or downregulation in the pairwise comparisons between 2-phenylethanol- or β-cyclocitral-treated compared to control leaf disks in *P. viticola*-inoculated samples and not modulated in mock-inoculated samples at 1 dpi or 6 dpi), and they were classified into 14 functional categories (columns AT), according to the manually curated annotation based on the protein descriptions (column AU) and Gene Ontology (GO) annotations (column AV) of grapevine genome annotation (*Vitis vinifera* PN40024.v4.1). Putative functions of DETs belonging to five functional categories possibly involved in grapevine resistance against downy mildew (defense, secondary metabolism, signal transduction, oxidative stress, and transcription) are reported in column AW.

Co-expression network analysis was carried out with a weighted gene co-expression network analysis (WGCNA; v1.72.1) using the Log_2 (RPM+1) values with a soft threshold power of 24 and a minimum of 15 transcripts per module, and it is indicated for each DET with the respective color (column AX) and code (column AY), are reported for each DET.

Table S6. Metabolic features detected by ultra high pressure liquid chromatography - heated electrospray ionization - Orbitrap mass spectrometry (UHPLC-HESI-Orbitrap-MS) analysis.

Grapevine leaf disks were treated with water (Control), 2-phenylethanol, or β-cyclocitral, inoculated with *Plasmopara viticola* (*P. viticola*-inoculated) or water (Mock-inoculated), and collected at one and six days post inoculation (dpi).

Detected metabolic features (column A), specified by mean mass to charge ratio (m/z; column B) and mean retention time (rt) expressed as minutes (column C), were detected by UHPLC-HESI-Orbitrap-MS analysis in negative heated-electrospray ionization (HESI) mode (blue cells in column A) and positive HESI mode (red cells in column A). Peak area (abundance) is reported for six replicates (pool of ten leaf disks each) of control mock-inoculated samples at 1 dpi (columns D-I), 2-phenylethanol-

treated mock-inoculated samples at 1 dpi (columns J-O), β-cyclocitral-treated mock-inoculated samples at 1 dpi (columns P-U), control *P. viticola*-inoculated samples at 1 dpi (columns V-AA), 2-phenylethanol-treated *P. viticola*-inoculated samples at 1 dpi (columns AB-AG), β-cyclocitral-treated *P. viticola*-inoculated samples at 1 dpi (columns AH-AM), control mock-inoculated samples at 6 dpi (columns AN-AS), 2-phenylethanol-treated mock-inoculated samples at 6 dpi (columns AT-AY), β-cyclocitral-treated mock-inoculated samples at 6 dpi (columns AZ-BE), control *P. viticola*-inoculated samples at 6 dpi (columns BF-BK), 2-phenylethanol-treated *P. viticola*-inoculated samples at 6 dpi (columns BL-BQ), β-cyclocitral-treated *P. viticola*-inoculated samples at 6 dpi (columns BR-BW). Quality control (QC) samples were obtained from the homogenization of equal aliquots of each sample (columns BX-CL).

Metabolic features were annotated with Compound Discoverer and the most probable chemical name (column CM), elemental formula (column CN), annotation delta mass (column CO), calculated molecular weight (column CP), fragmentation information (column CQ), neutral losses (column CR), reference ion (column CS), number of Chem Spider results (column CT), number of mzCloud results (column CU) are reported for each feature. No chemical name and/or chemical formula was found for metabolic features with empty cells of columns CM, CN, and CO.

Table S7 Annotated metabolic features detected by ultra high pressure liquid chromatography - heated electrospray ionization - Orbitrap mass spectrometry (UHPLC-HESI-Orbitrap-MS) analysis. Grapevine leaf disks were treated with water (Control), 2-phenylethanol or β-cyclocitral, inoculated with *Plasmopara viticola* (*P. viticola*-inoculated) or water (Mock-inoculated), and collected at one and six days post inoculation (dpi).

Metabolic features (column A), specified by mean mass to charge ratio (m/z; column B) and mean retention time (rt) expressed as minutes (column C), were detected by UHPLC-HESI-Orbitrap-MS analysis in negative heated-electrospray ionization (HESI) mode (blue cells in column A) and positive HESI mode (red cells in column A). Mean, standard error and coefficient of variation values of peak area (abundance) from six replicates (pool of ten leaf disks each) are reported for control mock-inoculated samples at 1 dpi (columns D-F), 2-phenylethanol-treated mock-inoculated samples at 1 dpi (columns G-I), β-cyclocitral-treated mock-inoculated samples at 1 dpi (columns J-L), control *P. viticola*-inoculated samples at 1 dpi (columns M-O), 2-phenylethanol-treated *P. viticola*-inoculated samples at 1 dpi (columns P-R), β-cyclocitral-treated *P. viticola*-inoculated samples at 1 dpi (columns S-U), control mock-inoculated samples at 6 dpi (columns V-X), 2-phenylethanol-treated mock-inoculated samples at 6 dpi (columns Y-AA), β-cyclocitral-treated mock-inoculated samples at 6 dpi (columns AB-AD), control *P. viticola*-inoculated samples at 6 dpi (columns AE-AG), 2-phenylethanol-treated *P. viticola*-inoculated samples at 6 dpi (columns AH-AJ), β-cyclocitral-treated *P. viticola*-inoculated samples at 6 dpi (columns AK-AM).

Metabolic features with significant increases (UP) or decreases (DOWN) in abundances were selected with Compound Discoverer, imposing a Log_2-transformed fold change lower than -1 or higher than 1 and a *P*-value of *t*-test lower than 0.05 in at least one pairwise comparison, such as between control *P. viticola*-inoculated and control mock-inoculated samples at 1 dpi (columns AN-AP), 2-phenylethanol-treated *P. viticola*-inoculated and 2-phenylethanol-treated mock-inoculated samples at 1 dpi (columns AQ-AS), 2-phenylethanol-treated mock-inoculated and control mock-inoculated samples at 1 dpi (columns AT-AV), 2-phenylethanol-treated *P. viticola*-inoculated and control *P. viticola*-inoculated samples at 1 dpi (columns AW-AY), β-cyclocitral-treated *P. viticola*-inoculated and β-cyclocitral-treated mock-inoculated samples at 1 dpi (columns AZ-BB), β-cyclocitral-treated mock-inoculated and control mock-inoculated samples at 1 dpi (columns BC-BE), β-cyclocitral-treated *P. viticola*-inoculated and control *P. viticola*-inoculated samples at 1 dpi (columns BF-BH), control *P. viticola*-inoculated and control mock-inoculated at 6 dpi (columns BI-BK), 2-phenylethanol-treated *P. viticola*-inoculated and 2-phenylethanol-treated mock-inoculated samples at 6 dpi (columns BL-BN), 2-phenylethanol-treated mock-inoculated and control mock-inoculated samples at 6 dpi (columns BO-BQ), 2-phenylethanol-treated *P. viticola*-inoculated and control *P. viticola*-inoculated samples at 6 dpi (columns BR-BT), β-cyclocitral-treated *P. viticola*-inoculated and β-cyclocitral-treated mock-inoculated samples at 6 dpi (columns BU-BW), β-cyclocitral-treated mock-inoculated and control mock-inoculated samples at 6 dpi (columns BX-BZ), β-cyclocitral-treated *P. viticola*-inoculated and control *P. viticola*-inoculated samples at 6 dpi (columns CA-CC).
Metabolic features with significant increases (UP) or decreases (DOWN) in abundance were selected with Compound Discoverer, imposing a Log_2-transformed fold change (LFC) lower than -1 or higher than 1 and a *P*-value of *t*-test lower than 0.05 in at least one pairwise comparison.
Metabolic features were annotated with Compound Discoverer and the most probable chemical name (column CD), elemental formula (column CE), annotation delta mass (column CF), calculated molecular weight (column CG), fragmentation information (column CH), neutral losses (column CI), reference ion (column CJ), number of Chem Spider results (column CK), number of mzCloud results (column CL) are reported for each feature.

Table S8. Functional annotations of metabolic features with significant changes in abundance in leaf disks treated with 2-phenylethanol.
Grapevine leaf disks were treated with water (Control) or 2-phenylethanol, inoculated with *Plasmopara viticola* (*P. viticola*-inoculated) or water (Mock-inoculated), and collected at one and six days post inoculation (dpi).
Metabolic features (column D), specified by mean mass to charge ratio (m/z; column E) and mean retention time (rt) expressed as minutes (column F) were detected using ultra high pressure liquid chromatography - heated electrospray ionization - Orbitrap mass spectrometry (UHPLC-HESI-

Orbitrap-MS) analysis in negative heated electrospray ionization (HESI) mode (blue cells in column D) and positive HESI mode (red cells in column D). Metabolic features were annotated with Compound Discoverer and the most probable chemical name (column G), elemental formula (column H), annotation delta mass (column I), calculated molecular weight (column J), fragmentation information (column K), neutral losses (column L), reference ion (column M), number of Chem Spider results (column N), number of mzCloud results (column O) are reported for each feature. Entry codes of Kyoto Encyclopedia of Genes and Genomes (KEGG) database were obtained with MetaboAnalyst imposing an error acceptance of 3 ppm (column P).

Metabolic features with significant increases (UP) or decreases (DOWN) in abundance were selected with Compound Discoverer, imposing a Log_2-transformed fold change (LFC) lower than -1 or higher than 1 and a *P*-value of *t*-test lower than 0.05 in at least one pairwise comparison (columns Q-AB), such as between control *P. viticola*-inoculated and control mock-inoculated samples, 2-phenylethanol-treated *P. viticola*-inoculated and 2-phenylethanol-treated mock-inoculated samples, 2-phenylethanol-treated mock-inoculated and control mock-inoculated samples, 2-phenylethanol-treated *P. viticola*-inoculated and control *P. viticola*-inoculated samples at 1 dpi and 6 dpi.

Annotated metabolic features with significant changes in abundance were grouped (group from 1 to 8; column C) according to the Venn diagrams (column A; Figure S7), in those modulated in one or two pairwise comparisons at 1 dpi and 6 dpi (column B). Metabolic pathway analysis was carried out using the pathway analysis tool of MetaboAnalyst based on the KEGG codes of annotated metabolic features with significant increases (groups 1, 2, 3, 4) or decreases (groups 5, 6, 7, 8) in abundance.

Manually curated annotation (columns AC-AM) was carried out for annotated metabolic features with LFC lower than -3 or higher than 3 and a *P*-value of *t*-test lower than 0.05 by searching all putative chemical names of each annotated feature found by Compound Discoverer in the PubChem (https://pubchem.ncbi.nlm.nih.gov/), ChEBI (https://www.ebi.ac.uk/chebi/), and KEGG (https://www.kegg.jp/kegg/compound/) databases, in order to retrieve the exact mass (column AE), considering the ionization mode of detection (column AF), InChI code (column AG), InChIKey code (column AH), and spectral information (database reference spectra). Additional *in silico* reference spectra were obtained with InChI codes in the CFM-ID 4.0 web server (https://cfmid.wishartlab.com). Database reference spectra and *in silico* reference spectra were visually compared with the experimental full scan MS spectra and ddMS2 fragmentation spectra of each annotated metabolic feature, in order to select the compound annotation (annotated compound; column AC), elemental formula (column AD), molecular ions (column AI), entry code of KEGG database (column AJ), and KEGG pathway (column AK).

Annotated compounds were classified into nine putative chemical classes according to the manually curated annotation (columns AL-AM) based on the classification obtained with the ClassyFire web-based application (https://cfb.fiehnlab.ucdavis.edu/) (columns AN-AS). Annotation level was

assigned according to the Metabolomics Standard Initiative (Sumner et al. 2007 doi: 10.1007/s11306-007-0082-2), such as identified metabolites (level 1), putatively annotated compounds (level 2), isomeric (level 2/3), putatively characterized compound classes (level 3), and unknown (level 4) (column AT).

Table S9. Metabolic pathway analysis results of annotated metabolic features with significant changes in abundances in leaf disks treated with 2-phenylethanol.
Grapevine leaf disks were treated with water (Control) or 2-phenylethanol, inoculated with *Plasmopara viticola* (*P. viticola*-inoculated) or treated with water (Mock-inoculated), and collected at one and six days post inoculation (dpi).
Metabolic features were detected with ultra high pressure liquid chromatography - heated electrospray ionization - Orbitrap mass spectrometry (UHPLC-HESI-Orbitrap-MS) analysis.
Metabolic features with significant changes in abundances were selected with Compound Discoverer, imposing a Log_2-transformed fold change (LFC) lower than -1 or higher than 1 and a *P*-value of *t*-test lower than 0.05 in at least one pairwise comparison, such as between control *P. viticola*-inoculated and control mock-inoculated samples, 2-phenylethanol-treated *P. viticola*-inoculated and 2-phenylethanol-treated mock-inoculated samples, 2-phenylethanol-treated mock-inoculated and control mock-inoculated samples, 2-phenylethanol-treated *P. viticola*-inoculated and control *P. viticola*-inoculated samples at 1 dpi and 6 dpi.
Metabolic pathway analysis was carried out using the pathway analysis tool of MetaboAnalyst based on the Kyoto Encyclopedia of Genes and Genomes (KEGG) codes of annotated metabolic features with changes in abundance and grouped (column A) in those with increases (groups 1, 2, 3, 4) or decreases (groups 5, 6, 7, 8) in abundance according to the Venn diagrams at 1 dpi and 6 dpi (Figure S7). Pathway name (column B), number of compounds in each pathway (column C), number of compounds expected by chance (column D), number of KEGG codes matched to each pathway (column E), and the raw *P*-value (column F) are calculated on the bases of the hypergeometric test. Log transformation (column G), Holm adjusted *P*-value (column H), false discovery rate (column I), and the impact obtained with the pathway topology analysis (column J) are reported for each pathway. Metabolic pathways with Holm adjusted *P*-value lower than 0.05 are highlighted in bold (adjusted *P*-value ≤ 0.05).

Table S10. Functional annotations of annotated metabolic features with significant changes in abundance in leaf disks treated with β-cyclocitral.
Grapevine leaf disks were treated with water (Control) or β-cyclocitral , inoculated with *Plasmopara viticola* (*P. viticola*-inoculated) or water (Mock-inoculated), and collected at one and six days post inoculation (dpi).

Metabolic features (column D), specified by mean mass to charge ratio (m/z; column E) and mean retention time (rt) expressed as minutes (column F) were detected using ultra high pressure liquid chromatography - heated electrospray ionization - Orbitrap mass spectrometry (UHPLC-HESI-Orbitrap-MS) analysis in negative heated electrospray ionization (HESI) mode (blue cells in column D) and positive HESI mode (red cells in column D). Metabolic features were annotated with Compound Discoverer and the most probable chemical name (column G), elemental formula (column H), annotation delta mass (column I), calculated molecular weight (column J), fragmentation information (column K), neutral losses (column L), reference ion (column M), number of Chem Spider results (column N), number of mzCloud results (column O) are reported for each feature. Entry codes of Kyoto Encyclopedia of Genes and Genomes (KEGG) database were obtained with MetaboAnalyst imposing an error acceptance of 3 ppm (column P).

Metabolic features with significant increases (UP) or decreases (DOWN) in abundance were selected with Compound Discoverer, imposing a Log_2-transformed fold change (LFC) lower than -1 or higher than 1 and a *P*-value of *t*-test lower than 0.05 in at least one pairwise comparisons (columns Q-AB), such as between control *P. viticola*-inoculated and control mock-inoculated samples, β-cyclocitral-treated *P. viticola*-inoculated and β-cyclocitral-treated mock-inoculated samples, β-cyclocitral-treated mock-inoculated and control mock-inoculated samples, β-cyclocitral-treated *P. viticola*-inoculated and control *P. viticola*-inoculated samples at 1 dpi and 6 dpi.

Annotated metabolic features with significant changes in abundance were grouped (group from 9 to 16; column C) according to the Venn diagrams (column A; Figure S8), in those modulated in one or two pairwise comparisons at 1 dpi and 6 dpi (column B). Metabolic pathway analysis was carried out using the pathway analysis tool of MetaboAnalyst based on the KEGG codes of annotated metabolic features with significant increases (groups 9, 10, 11, 12) or decreases (groups 13, 14, 15, 16) in abundance.

Manually curated annotation (columns AC-AM) was carried out for annotated metabolic features with LFC lower than -3 or higher than 3 and a *P*-value of *t*-test lower than 0.05 by searching all putative chemical names of each annotated feature found by Compound Discoverer in the PubChem (https://pubchem.ncbi.nlm.nih.gov/), ChEBI (https://www.ebi.ac.uk/chebi/), and KEGG (https://www.kegg.jp/kegg/compound/) databases, in order to retrieve the exact mass (column AE), considering the ionization mode of detection (column AF), InChI code (column AG), InChIKey code (column AH), and spectral information (database reference spectra). Additional *in silico* reference spectra were obtained with InChI codes in the CFM-ID 4.0 web server (https://cfmid.wishartlab.com). Database reference spectra and *in silico* reference spectra were visually compared with the experimental full scan MS spectra and ddMS2 fragmentation spectra of each annotated metabolic feature, in order to select the compound annotation (annotated compound; column AC), elemental

formula (column AD), molecular ions (column AI), entry code of KEGG database (column AJ), and KEGG pathway (column AK).

Annotated compounds were classified into nine putative chemical classes according to the manually curated annotation (columns AL-AM) based on the classification obtained with the ClassyFire web-based application (https://cfb.fiehnlab.ucdavis.edu/) (columns AN-AS). Annotation level was assigned according to the Metabolomics Standard Initiative (Sumner et al. 2007 doi: 10.1007/s11306-007-0082-2) such as identified metabolites (level 1), putatively annotated compounds (level 2), isomeric (level 2/3), putatively characterized compound classes (level 3), and unknown (level 4) (column AT).

Table S11. Metabolic pathway analysis results of annotated metabolic features with significant changes in abundances in leaf disks treated with β-cyclocitral.

Grapevine leaf disks were treated with water (Control) or β-cyclocitral, inoculated with *Plasmopara viticola* (*P. viticola*-inoculated) or water (Mock-inoculated), and collected at one and six days post inoculation (dpi).

Metabolic features were detected with ultra high pressure liquid chromatography - heated electrospray ionization - Orbitrap mass spectrometry (UHPLC-HESI-Orbitrap-MS) analysis. Metabolic features with significant changes in abundances were selected with Compound Discoverer, imposing a Log_2-transformed fold change (LFC) lower than -1 or higher than 1 and a *P*-value of *t*-test lower than 0.05 in at least one pairwise comparison, such as between control *P. viticola*-inoculated and control mock-inoculated samples, β-cyclocitral-treated *P. viticola*-inoculated and β-cyclocitral-treated mock-inoculated samples, β-cyclocitral-treated mock-inoculated and control mock-inoculated samples, β-cyclocitral-treated *P. viticola*-inoculated and control *P. viticola*-inoculated samples at 1 dpi and 6 dpi.

Metabolic pathway analysis was carried out using the pathway analysis tool of MetaboAnalyst based on the Kyoto Encyclopedia of Genes and Genomes (KEGG) codes of annotated metabolic features with changes in abundance and grouped (column A) in those with increases (groups 9, 10, 11, 12) or decreases (groups 13, 14, 15, 16) in abundance according to the Venn diagrams at 1 dpi and 6 dpi (Figure S8). Pathway name (column B), number of compounds in each pathway (column C), number of compounds expected by chance (column D), number of KEGG codes matched to each pathway (column E), and the raw *P*-value (column F) are calculated on the bases of the hypergeometric test. Log transformation (column G), Holm adjusted *P*-value (column H), false discovery rate (column I) and the impact obtained with the pathway topology analysis (column J) are reported for each pathway. Metabolic pathways with Holm adjusted *P*-value lower than 0.05 are highlighted in bold (adjusted *P*-value ≤ 0.05).

Table S12. Correlation of gene co-expression modules of differentially expressed transcripts with the annotated compounds.

A. Co-expression network analysis of differentially expressed transcripts (DETs) was carried out with a weighted gene co-expression network analysis (WGCNA; v1.72.1) using the Log_2 (RPM+1) values with a soft threshold power of 24 and a minimum number of transcripts per module of 15 (Figure S10). WGCNA was used to calculate the correlation of each co-expression module, indicated with the respective color (column A) and number (column B), with the 198 annotated compounds (column C), according to a Pearson's correlation method (columns D-F). For each annotated compound, the chemical name (column G), KEGG code (column H), manually curated annotation (column I), elemental formula (column J), and chemical class (column K) are reported.

B. List of 198 annotated compounds with significant changes in abundance (LFC lower than -3 or higher than 3 and a *P*-value of t-test lower than 0.05) and classified into putative chemical classes (benzenoids, carbohydrates and conjugates, carbonyl compounds, carboxylic acids and derivatives, indoles and derivatives, lipids and lipid-like compounds, phenylpropanoids, terpenoids, and unknown) by the manually curated annotation.
